# Poly(bromocresol green) and poly(bromocresol purple) films by electropolymerization in ternary deep eutectic solvent on gold nanoparticle/graphene quantum dot modified electrodes for the biosensing of glucose

**DOI:** 10.1007/s00216-026-06524-z

**Published:** 2026-05-04

**Authors:** Joseany M. S. Almeida, Christopher M. A. Brett

**Affiliations:** Department of Chemistry, CEMMPRE, ARISE, Faculty of Sciences and Technology, University of Coimbra, 3004-535 Coimbra, Portugal

**Keywords:** Ternary deep eutectic solvent, Electropolymerization, Poly(bromocresol green) and poly(bromocresol purple), Gold nanoparticles and graphene quantum dots, Glucose electrochemical biosensor, Wine samples

## Abstract

The triarylmethane dyes brilliant cresyl green (BCG) and brilliant cresyl purple (BCP) have been electropolymerized on glassy carbon electrodes (GCE) modified with gold nanoparticles (AuNP) and graphene quantum dots (GQD) dispersed in chitosan (AuNP+GQD)_chitosan_ by potential cycling in a novel ternary deep eutectic solvent (tDES). The tDES was composed of choline chloride (ChCl) as the hydrogen bond acceptor plus acetic acid and ethylene glycol as hydrogen bond donors, with perchloric acid as a tDES acid dopant. GQD_chitosan_ was synthesized by a simple and eco-friendly procedure using citric acid as the carbon source in chitosan solution with 1% v/v acetic acid. AuNP dispersed in citrate buffer were mixed with GQD_chitosan_ in the proportion (70/30% v/v). The PBCG/(AuNP+GQD)_chitosan_/GCE and PBCP/(AuNP+GQD)_chitosan_/GCE modified electrodes were compared and characterized by cyclic voltammetry and electrochemical impedance spectroscopy. The morphology of the synthesized polymer films was examined by scanning electron microscopy. After immobilization of glucose oxidase (GOx), GOx/PBCG/(AuNP+GQD)_chitosan_/GCE and GOx/PBCP/(AuNP+GQD)_chitosan_/GCE biosensors were employed for the determination of glucose by amperometry at a potential of −0.2 V vs. Ag/AgCl. Linear concentration ranges were 10–200 µM for GOx/PBCG/(AuNP+GQD)_chitosan_/GCE and 10–140 µM for GOx/PBCP/(AuNP+GQD)_chitosan_/GCE, with corresponding limits of detection of 2.3 µM and 3.1 µM. The biosensor with PBCG film showed slightly better analytical parameters. The biosensors demonstrated good repeatability, reproducibility, stability, and selectivity and were successfully applied to wine samples with very good recoveries.

**Supplementary Information:**

The online version contains supplementary material available at 10.1007/s00216-026-06524-z.

## Introduction

The preparation of modified electrodes in deep eutectic solvents (DES) is a topic that has been growing in importance in recent years. DES are composed of hydrogen bond acceptors (HBA) and hydrogen bond donors (HBD) capable of associating and forming a eutectic phase, characterized by having a melting point lower than that of its components (below 100 °C) [1]. The type III DES used by us is formed by a quaternary ammonium salt as HBA and one or two HBD with hydroxy groups. A binary DES is formed by the combination of one HBA and one HBD, and the addition of a second HBD results in a ternary DES [1]. The advantages of employing DES are their cost-effectiveness, ease of preparation, low toxicity, biodegradability, and high solubility.

In the electrochemical context, binary and ternary DES have been most widely utilized in the electropolymerization of redox monomers to prepare modified electrode sensors and biosensors [2–4]. Redox polymer films formed from phenazine dyes have been associated with carbon nanomaterial modifiers such as multiwalled carbon nanotubes (MWCNT) [2, 5], graphene (GR) [6, 7], reduced graphene oxide (rGO) [8, 9], carbon quantum dots (CQD) [10, 11], and graphene quantum dots (GQD) as well as with metallic nanoparticles [12], due to their catalytic ability, synergic effects, enhancement of selectivity, and sensitivity [13].

GQD combined with gold nanoparticles (AuNP) can be employed to promote the formation of redox polymer films by electropolymerization in sensor preparation. GQD are isolated sheets of graphene with small dimensions (less than 100 nm) that produce intense photoluminescence due to quantum confinement [14, 15] and have been utilized in analytical sensors, e.g. [13]. AuNP have been extensively used for chemical and biological sensing applications, particularly regarding nanocomposites of AuNP plus conducting polymers, due to their excellent electronic, magnetic, optical, and catalytic properties, as well as characteristics arising from surface functionalization [12]. Gold surfaces have a high affinity for thiol (–SH) and amine (–NH_2_) groups, which can help bind biomolecules and thence they can be used for biomarker sensing [16].

Electroactive redox polymers are used in electrochemical enzyme biosensors due to their enhancement of the electrochemical signals generated by the redox reactions of target compounds [3, 4, 17]. Bromocresol green (BCG) and bromocresol purple (BCP) are triphenylmethane dyes that have similar structures (see Scheme S1), the main differences being that BCG has more bromine atoms and methyl groups. They have been used as modifier electrode materials in electrochemical sensors [18–21]. Besides good conductivity, their high-electron-density hydroxyl groups give PBCG and PBCP films a negative charge, and they exhibit electrocatalytic activity towards many substances. For example, a PBCG film-modified electrode was prepared with PBCG associated with Fe_3_O_4_ and MWCNT; interaction between the target analytes and the electrode led to a detection limit (LOD) of 80 nM for determining serotonin [18]. The simultaneous determination of uric acid, dopamine, and ascorbic acid was carried out at a PBCG-modified glassy carbon electrode (GCE) with LODs of 0.17, 0.017, and 0.17 µM, respectively, in human serum samples and vitamin C tablets [19]. A MWCNT and PBCP modified carbon paste electrode (MWCNT_ox_/PBCP/MCPE) was prepared to determine l-tyrosine with an LOD of 1.91 × 10^–7^ M in spiked milk and blood serum samples [20]. In other work, the green synthesis of a PBCP/graphene composite was carried out and the modified electrode was applied to the electrochemical determination of 2,4,6-trichlorophenol with an LOD of 5.0 × 10^–9^ M in water samples [21].

Glucose is a monosaccharide that is important in cells as an energy source and an intermediate metabolite. Its accurate determination is important for controlling its level in the blood, particularly in the context of diabetes [4]. Glucose also plays an important role in the quality control of technological processes and food products, such as wine. Usually, the determination of glucose is made with glucose oxidase (GOx) enzyme in an enzyme biosensor, as reported in the literature [17, 22, 23].

In this work, the electropolymerization of BCG and BCP on GCE previously modified by drop-casting with AuNP plus GQD dispersed in chitosan was carried out for the first time by potential cycling in a novel ternary DES (choline chloride (ChCl) as HBA with acetic acid (AcA) and ethylene glycol (EG) as HBDs). This exploits the interaction between AuNP and GQD, which are synthesized in a simple and eco-friendly procedure and are demonstrated to lead to higher sensitivity. The new modified electrode platforms were characterized by electrochemical voltammetric techniques, electrochemical impedance, and scanning electron microscopy (SEM). GOx was then immobilized on the PBCG and PBCP modified electrodes to develop enzymatic biosensors for glucose detection by fixed potential amperometry. The analytical parameters of the two biosensors were compared, given the similarities in the monomer chemical structure, mainly methyl substituents in the BCG ring being replaced by bromines in BCP, to probe any effects from the use of PBCP compared with PBCG triarylmethane polymers. Application to the determination of glucose in wine was demonstrated.

## Experimental

### Reagents and solutions

All reagents were of analytical grade and were utilized as supplied, without further purification. Glucose oxidase (GOx, E.C. 1.1.3.4, from *Aspergillus niger*, 24 U/mg) was acquired from Fluka, Switzerland. αD(+)-glucose, glutaraldehyde (GA) (25% v/v in water), and bovine serum albumin (BSA) were purchased from Sigma, Germany. Choline chloride, ethylene glycol (anhydrous, 99.8%), sulfuric acid (H_2_SO_4_, 98%), nitric acid (HNO_3_, 65%), perchloric acid (HClO_4_, 70%), hydrochloric acid (HCl, 36.5%), acetic acid (CH_3_COOH, 99.99%), sodium hydroxide (NaOH), chitosan from crab shells (deacetylated > 85%), catechol (CC), fructose (Fru), uric acid (UA), ascorbic acid (AA), citric acid (CA), tartaric acid (TTA), acetaminophen (APAP), and lactic acid (LA) were supplied by Sigma-Aldrich, Germany. Bromocresol green monomer (BCG) (C_21_H_14_Br_4_O_5_S) and bromocresol purple monomer (BCP) (C_21_H_16_Br_2_O_5_S), structures shown in Scheme S1, were from Sigma-Aldrich, Germany. Boric acid was from Baker, UK, and phosphoric acid was from Riedel-de Haën, Switzerland. Gold nanoparticles (AuNP) (20 nm diameter, 6.54 × 10^11^ nanoparticles/mL and stabilized in citrate buffer) were supplied by Sigma-Aldrich.

Solutions of 1 mM BCG and BCP were prepared in ethaline DES (1:2 molar ratio) with 1 M HClO_4_ as dopant acid. BCG and BCP solutions, concentration 1 mM, were also prepared in ChCl:AcA:EG tDES (1:2:2 molar ratio), with the addition of different dopant acids, namely H_2_SO_4_, HNO_3_, HClO_4_, and HCl.

Britton-Robinson (BR) buffer solutions were prepared by mixing phosphoric acid, acetic acid, and boric acid (all solutions of 0.1 M concentration). Phosphate buffer saline (NaPBS) solutions were prepared by dissolving 3.450 g of monobasic sodium phosphate (NaH_2_PO_4_.H_2_O), 4.450 g of dibasic sodium phosphate (Na_2_HPO_4_.2H_2_O) plus 0.731 g of NaCl in 250 mL of Milli-Q water, final concentrations 0.10 M, 0.10 M, and 0.05 M, respectively. All buffer solutions had the pH adjusted with 10 M sodium hydroxide solution.

Chitosan solution (1.0% w/v) was made by dissolving a mass of 100 mg of chitosan powder in 10 mL of 1.0% v/v acetic acid solution with stirring.

Stock solutions of 0.10 M and 1.0 mM glucose were prepared in NaPBS every day.

All solutions were prepared using Millipore Milli-Q nanopure water (resistivity ≥ 18 MΩ cm), and all experiments were conducted at room temperature (25 ± 1 °C).

### Instrumentation

An Ivium CompactStat potentiostat (Ivium Technologies, Netherlands) controlled by IviumSoft software (version 2.024) was utilized to conduct voltammetric experiments in a conventional three-electrode cell containing a GCE (diameter 1 mm, geometric area 0.00785 cm^2^) (EDAQ, Australia) as the working electrode, a platinum wire as the auxiliary electrode, and Ag/AgCl (3 M KCl) as the reference electrode. All current responses are normalized to the GCE geometric area.

Electrochemical impedance spectroscopy (EIS) measurements were performed using a Solartron 1250 frequency response analyzer, coupled to a Solartron 1286 Electrochemical Interface, with ZPlot 2.4 software (Solartron Analytical, Ametek, UK). A sinusoidal voltage perturbation of amplitude 10 mV rms was applied in the frequency range between 65 kHz and 0.1 Hz, with 10 frequency steps per decade. ZView software (version 2.4, Scribner Associates, USA) was utilized to fit the spectra to equivalent electrical circuits.

The morphological characterization of the electrodes was carried out using scanning electron microscopy (SEM) with a JEOL instrument, JSM-5310, Japan, on carbon film electrodes (CFE) bare or modified with (AuNP+GQD)_chitosan_ and without or with a polymer film. These carbon film electrodes were made from carbon film electrical resistors with an exposed area of 0.020 cm^2^, as in Pedro et al. [2].

Solution pH measurements were made with an Edge pH-meter (HANNA Instruments, Woonsocket, RI, USA).

### Pre-treatment of GCE, preparation of GQD_chitosan_ and AuNP_chitosan_, and modification of GCE surface

Prior to modification, the bare GCE was carefully polished with 3 µm particle size diamond spray (Kemet, UK) on a polishing pad and rinsed with Milli-Q water.

The pyrolysis of citric acid to produce GQD_chitosan_ in 1% w/v chitosan solution dissolved in 1.0% v/v of acetic acid was done following a literature procedure with minor modifications [13]. First, 0.5 g of citric acid was heated to 240 °C. When the mixture became molten (with color changing from colorless to pale yellow and then to brown) after 2–5 min, the hot liquid containing GQD was added to 5.0 mL of chitosan solution (1.0% w/v) and stirred at room temperature for 10 min to obtain a clear pale-yellow dispersion of GQD_chitosan_, loading corresponding to 37.5 mg mL^−1^.

AuNP (20 nm diameter, 6.54 × 10^11^ nanoparticles/mL and stabilized in citrate buffer) were used to prepare a dispersion of AuNP_chitosan_ at a concentration of 4.58 × 10^11^ nanoparticles/mL in 0.43% chitosan dissolved in 1.0% v/v acetic acid, as previously reported by Abad-Gil and Brett [12]. The chitosan guarantees a stable and adherent AuNP layer on the working electrode surface.

A dispersion of (AuNP+GQD)_chitosan_,_1%w/v_ with a concentration of 4.58 × 10^11^ nanoparticles/mL of AuNP in GQD_chitosan_,_1%w/v_ was prepared by mixing 70/30 v/v of AuNP and GQD_chitosan_,_1%w/v_ [12], followed by sonication in an ultrasonic bath for 2 h, to obtain a homogeneous dispersion. This dispersion was kept under refrigeration and homogenized before use.

The bare GCE was modified, in the optimized procedure, by drop-casting 4.0 μL (2 × 2 μL) of AuNP_chitosan_, GQD_chitosan_, or (AuNP+GQD)_chitosan_ dispersion onto the electrode surface and drying in air at room temperature for 60 min. The AuNP_chitosan_ or (AuNP+GQD)_chitosan_ modified electrode was then activated by potential cycling between −0.6 V and 0.8 V vs. Ag/AgCl for 5 cycles in 0.1 M BR buffer solution, pH 3.0. The activation was performed to remove part of the capping agent and to promote polymer nucleation on the nanoparticles; without this procedure, nucleation does not occur, and there is no electropolymerization (see [12]). After this, the electrodes were ready for use.

### Preparation of ethaline and ternary DES

Ethaline DES was prepared by mixing HBA (ChCl) and HBD (EG) in a molar ratio of 1:2, designated as ChCl:EG. A ternary DES (tDES) mixture was prepared by mixing HBA (ChCl) with the HBDs, acetic acid (AcA), and EG, in a molar ratio of 1:2:2, designated as ChCl:AcA:EG [5]. To prepare ethaline DES and a tDES, first, ChCl was heated for 5 min to evaporate any water, followed by the addition of the HBDs, EG for ethaline DES and AcA and EG for tDES. The mixtures were maintained at 60 °C under mechanical stirring until a homogeneous solution was achieved. The ethaline and tDES were cooled down to room temperature and were then ready to use.

### Electropolymerization and characterization of PBCG/(AuNP+GQD)_chitosan_/GCE and PBCP/(AuNP+GQD_chitosan_)/GCE in ethaline and ternary DES

Electropolymerization of the monomers BCG and BCP (concentration 1.0 mM) was first carried out by potential cycling in ethaline DES (1:2) at (AuNP+GQD)_chitosan_/GCE with 1 M HClO_4_ as acid dopant. The ternary DES ChCl:AcA:EG was then evaluated for polymerization on GCE, AuNP_chitosan_/GCE, and (AuNP+GQD)_chitosan_/GCE. Four acid dopants, HNO_3_, H_2_SO_4_, HClO_4_, and HCl, at a concentration in the monomer solution of 1.0 M, were tested, being added directly to the polymerization solution.

For PBCG, the optimized potential cycling range was −0.6 V to + 1.3 V vs. Ag/AgCl and for PBCP it was −0.6 V to + 1.2 V vs. Ag/AgCl at a scan rate of 50 mV s^−1^ for 15 cycles, starting at the negative potential limit. The polymer films were characterized in 0.1 M BR aqueous solution, at pH 3.0, as in previous work with other electroactive dyes polymerized in DES [5], by cyclic voltammetry (CV) in the potential range, −0.6 to + 1.4 V vs. Ag/AgCl for PBCG and −0.5 V to + 1.0 V vs. Ag/AgCl for PBCP, in the scan rate range 10–100 mV s^−1^, starting at the negative potential limit.

### Preparation of the biosensors

GOx was immobilized on PBCG/(AuNP+GQD)_chitosan_/GCE and PBCG/(AuNP+GQD)_chitosan_/GCE prepared in tDES [17]. Enzyme immobilization was performed using the cross-linking reaction with glutaraldehyde (GA) and bovine serum albumin (BSA). A mixture containing 5 µL of GA (2.5% v/v in water) and 10 µL of enzyme solution was used. The latter was prepared by dissolving 40 mg BSA and 10 mg GOx in 1 mL of 0.1 M NaPBS (pH 7.0). For drop-coating, 3 µL of the mixture described before was placed onto the electrode previously modified with one of the polymers (PBCG or PBCP). The electrode assembly was left to dry at room temperature for at least 1 h after which the biosensor could be used immediately. When not in use, the electrode was kept at 4 °C in NaPBS (pH 7.0).

### Analytical procedure for glucose, interferences, and sample preparation

Glucose was determined at GOx/PBCG/(AuNP+GQD)_chitosan_/GCE and GOx/PBCP/(AuNP+GQD)_chitosan_/GCE by fixed potential amperometry at −0.2 V vs. Ag/AgCl in 0.1 M NaPBS pH 7.0, as in Ghica and Brett [17]. All measurements were performed in triplicate. Calibration curves in the concentration range 10 to 200 µM for GOx/PBCG/(AuNP+GQD)_chitosan_/GCE and 10 to 140 µM for GOx/PBCP/(AuNP+GQD)_chitosan_/GCE were constructed from the current response following injection of aliquots (20 µL) of a standard solution of 1 mM glucose into a volume of 2 mL of 0.1 M NaPBS pH 7.0 in the electrochemical cell.

Interference studies were made at GOx/PBCG/(AuNP+GQD)_chitosan_/GCE and GOx/PBCP/(AuNP+GQD)_chitosan_/GCE sensors using the same experimental methodology and conditions. The potential interferents catechol (CC), fructose (Fru), uric acid (UA), ascorbic acid (AA), citric acid (CA), tartaric acid (TTA), acetaminophen (APAP), and lactic acid (LA) were evaluated. The interferents were selected based on substances that can be present in fruits or foods. Lactic acid is used as a preservative and acidulant in products such as yogurt and cheese, helping maintain their quality and shelf life. Tartaric acid is a substance present in grapes and wine and is an acid regulator of wine. Uric acid can be present in samples of urine or blood, and acetaminophen is a common medication. To this end, a potential of −0.2 V vs. Ag/AgCl was applied and injections of 40 µL of a 1 mM glucose standard were made into a volume of 2 mL of 0.1 M NaPBS pH 7.0 before and after adding the interferents. The added interferents (40 µL of each) were all prepared with a concentration of 1 mM.

Glucose was determined in red wine and white wine, acquired in a supermarket in Coimbra, Portugal. The contents of glucose in the samples were determined by amperometry at a fixed potential of −0.2 V vs. Ag/AgCl, as described above, using the standard addition method. To prepare the wine samples, a volume of 500 µL was diluted in 25 mL of 0.1 M NaPBS (pH 7.0). Then, an appropriate volume (~40 µL of sample) was injected into the electrochemical cell containing 2 mL of 0.1 M NaPBS (pH 7.0), followed by additions of standard.

## Results and discussion

### Polymerization of BCG and BCP on bare GCE, AuNP_chitosan_/GCE, GQD_chitosan_/GCE and (AuNP+GQD)_chitosan_/GCE in ternary DES

A preliminary study of the potential cycling electropolymerization of BCG and BCP prepared in ChCl:AcA:EG tDES (1:2:2 molar ratio) at (AuNP+GQD)_chitosan_/GCE, using H_2_SO_4_ as the acid dopant, was carried out (see Fig. S1) for 15 cycles at a scan rate of 50 mV s^−1^. Different positive potential limits were tested, namely +1.0 V, +1.1 V, +1.2 V, +1.3 V, and +1.4 V vs. Ag/AgCl. Comparison of the response of the polymer-modified electrodes in 0.1 M BR pH 3.0 for PBCG and PBCP is shown in Fig. S1A and Fig. S1B, respectively. For PBCG, the highest current peak currents *j*_p_(II_a_)_PBCG_ = 28.2 ± 0.04 µA cm^−2^ were obtained when +1.3 V vs. Ag/AgCl was used as the positive potential limit. For PBCP, the highest current, peak, *j*_p_(II_a_)_PBCP_ = 19.9 ± 0.02 µA cm^−2^, was observed with a limit of +1.2 V vs. Ag/AgCl. For this reason, the positive limits of the potential range for electropolymerization in all further experiments were defined as −0.6 V to +1.3 V vs. Ag/AgCl for BCG, and −0.6 V to + 1.2 V vs. Ag/AgCl for BCP.

**Fig. 1:**
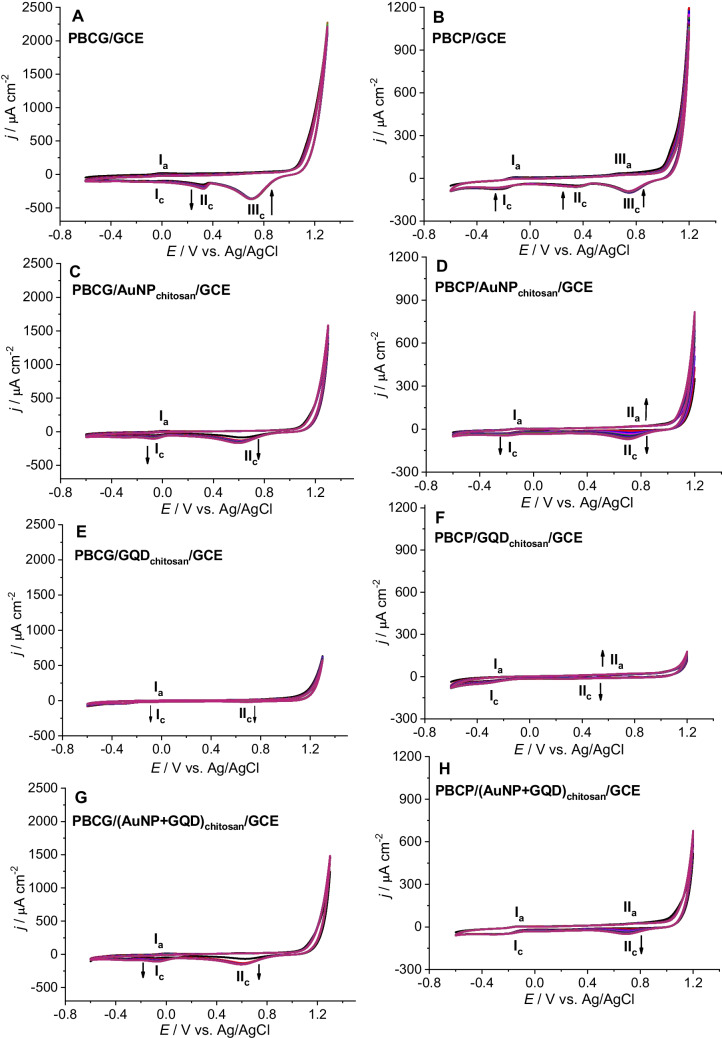
Electropolymerization of 1 mM BCG and 1 mM BCP in tDES (ChCl:AcA:EG 1:2:2 molar ratio) plus 1 M H_2_SO_4_ acid dopant by potential cycling for 15 cycles at 50 mV s^−1^: **A**, **B** bare GCE. **C**, **D** AuNP_chitosan_/GCE. **E**, **F** GQD_chitosan_/GCE. **G**, **H** (AuNP+GQD)_chitosan_/GCE

Electropolymerization of BCG and BCP, prepared in ChCl:AcA:EG tDES with molar ratio 1:2:2, was then carried out on bare GCE, AuNP_chitosan_/GCE, GQD_chitosan_/GCE, and (AuNP+GQD)_chitosan_/GCE by potential cycling for 15 cycles at a scan rate of 50 mV s^−1^ (see Fig. 1), utilizing 1.0 M H_2_SO_4_ acid dopant to lower the viscosity and increase the conductivity of tDES. Increasing the ionic strength of the solution also increases the rate of diffusion of species and makes the initiation of the polymerization process easier via cation radicals formed, beginning in the first cycle of electropolymerization [17].

For BCG electropolymerization on GCE, the response presented one redox couple I_a_/I_c_ (~ −0.005 V/~ −0.03 V) vs. Ag/AgCl, and two separate cathodic peaks in the region II_c_ (~0.3 V vs. Ag/AgCl) and III_c_ (~0.7 V vs. Ag/AgCl), respectively (Fig. 1A). The current peak increased only for peak II_a_, indicating that the growth of the polymer film was poor. For BCP electropolymerization, the CV response shows two redox couples, I_a_/I_c_ (~ −0.10 V/~ −0.20 V) vs. Ag/AgCl, III_a_/III_c_ (~0.66 V/~0.75 V) vs. Ag/AgCl, and a reduction peak with no visible peak II_c_ (~0.35 V) vs. Ag/AgCl (Fig. 1B). Formation of the PBCP polymer film was unsuccessful because the current peak decreased with an increase in the number of cycles.

PBCG and PBCP films were then formed by electropolymerization on AuNP-modified GCE, after activation. As reported in the literature, depending on the type of metallic or metal oxide nanoparticles, such an activation step may be required before electropolymerization [12]. The purpose of the activation procedure is to remove the capping agent from the AuNP, enabling electrostatic interaction between the monomer/polymer and the nanoparticle, facilitating the nucleation and growth of the polymer films on the nanoparticle. The AuNP prepared in a 1% chitosan solution were drop-cast on the surface of the GCE (2 µL (2 times)). After drying at room temperature, the electrode was immersed in 0.1 M BR buffer at pH 3. Then activation was accomplished by cycling 5 times at 50 mV s^−1^ between −0.6 and +0.8 V vs. Ag/AgCl. Following complete drying, electropolymerization on AuNP_chitosan_/GCE was carried out in the same way as at bare GCE (Fig. 1C and D). For both PBCG and PGCP, the growth of the polymer film was effective.

GCE was also modified with GQD_chitosan_ (2 µL, 2 times) and the response for electropolymerization is presented in Fig. 1E and F. For both dyes, the amount of polymer film growth was lower than that on AuNP_chitosan_/GCE.

Following this, the GCE was modified with both AuNP and GQD-chitosan before electropolymerization, i.e., on (AuNP+GQD)_chitosan_/GCE (see Fig. 1G and H), for PBCG and PBCP, respectively. For PBCG, the electropolymerization presented one redox couple (I_a_/I_c_) at (~ −0.009 V/~ −0.07 V) vs. Ag/AgCl and one cathodic peak (II_c_) at (~0.59 V) vs. Ag/AgCl. For PBCP, there are two redox couples (I_a_/I_c_) at (~ −0.13 V/~ −0.27 V) vs. Ag/AgCl and (II_a_/II_c_) at (~0.75 V/~0.69 V) vs. Ag/AgCl. The combination of AuNP plus GQD led to an increase in the amount of growth of both polymer films, and evaluation in 0.1 M BR pH 3 demonstrated the best response with higher current peaks, as can be seen in Fig. S2A and Fig. S2B.

The peak current densities for PBCG and PBCP on bare GCE *j*_p_(II_a_)_PBCG_ = 30.9 ± 0.02 µA cm^−2^ and *j*_p_(II_a_)_PBCP_ = 14.6 ± 0.03 µA cm^−2^, AuNP_chitosan_/GCE *j*_p_(II_a_)_PBCG_ = 25.1 ± 0.19 µA cm^−2^ and *j*_p_(II_a_)_PBCP_ = 13.8 ± 0.22 µA cm^−2^, and GQD_chitosan_/GCE *j*_p_(II_a_)_PBCG_ = 33.2 ± 0.11 µA cm^−2^ and *j*_p_(II_a_)_PBCP_ = 14.1 ± 0.10 µA cm^−2^ were lower than when using (AuNP+GQD)_chitosan_/GCE *j*_p_(II_a_)_PBCG_ = 42.4 ± 0.15 µA cm^−2^ and *j*_p_(II_a_)_PBCP_ = 19.9 ± 0.24 µA cm^−2^, demonstrating that the combination with AuNP and GQD improves the growth of the polymer films. The amount of PBCG polymer film was greater than that of PBCP, based on the peak current density values obtained in 0.1 M BR buffer, pH 3, aqueous medium, showing influence of the monomer structure on the polymer films, namely the presence of methyl vs. bromine substituents in the aromatic rings of BCG and BCP. Electropolymerization of BCG and BCP in ethaline DES leads to a polymer-modified electrode with lower currents in BR buffer than if tDES is used. In aqueous medium (0.1 M NaOH plus 0.1 M HClO_4_) (see Fig. S3A and Fig. S3B), the electropolymerization of BCG and BCP at (AuNP+GQD)_chitosan_/GCE was not effective for polymer film growth, with decreases in the peak currents with increasing number of cycles.

### Effect of scan rate and acid dopant on electropolymerization

The influence of scan rate and acid dopant identity on the electropolymerization of PBCG and PBCP was investigated in the tDES.

Polymerizations were carried out with scan rates in the range 20 to 200 mV s^−1^ (see Fig. S4 (A to E) for PBCG and Fig. S5 (A to E) for PBCP). The electrochemical characteristics of the resultant polymer film-modified electrodes, PBCG/(AuNP+GQD)_chitosan_/GCE and PBCP/(AuNP+GQD)_chitosan_/GCE, were studied in 0.1 M BR solution at pH 3, shown in Fig. S4F and Fig. S5F, respectively. For PBCG, the highest anodic peak current density of *j*_p_(II_a_) = 32.3 ± 0.02 µA cm^−2^ was obtained using a 50 mV s^−1^ scan rate, being lower at 20 mV s^−1^ *j*_p_(II_a_) = 27.9 ± 0.03 µA cm^−2^, 100 mV s^−1^ *j*_p_(II_a_) = 23.1 ± 0.05 µA cm^−2^, 150 mV s^−1^ *j*_p_(II_a_) = 17.3 ± 0.03 µA cm^−2^, and 200 mV s^−1^ *j*_p_(II_a_) = 10.1 ± 0.02 µA cm^−2^. Also for PBCP, the highest anodic peak current density *j*_p_(II_a_) = 19.9 ± 0.02 µA cm^−2^ was again obtained using a 50 mV s^−1−^scan rate.

The value at 20 mV s^−1^ was *j*_p_(II_a_) = 16.2 ± 0.01 µA cm^−2^, at 100 mV s^−1^ *j*_p_(II_a_) = 16.3 ± 0.03µA cm^−2^, at 150 mV s^−1^ *j*_p_(II_a_) = 9.5 ± 0.02 µA cm^−2^, and at 200 mV s^−1^ *j*_p_(I_a_) = 8.6 ± 0.02 µA cm^−2^. This is in agreement with previous work in viscous DES solutions, due to limitations on monomer diffusion to the electrode surface, such that the monomer is not replenished at the electrode surface sufficiently fast [2]. A scan rate of 50 mV s^−1^ was thus selected to prepare both polymer films.

Potential cycling electropolymerization for BCG and PBCP was also performed using different acid dopants, HClO_4_, HCl, HNO_3_, and H_2_SO_4_, all of concentration 1 M, primarily added to enhance the conductivity of the tDES. The polymer films were made by potential cycling in the potential range −0.6 to +1.3 V vs. Ag/AgCl for PBCG and −0.6 to +1.2 V vs. Ag/AgCl for PBCP, at a scan rate of 50 mV s^−1^ (Fig. S6 (A to D) and Fig. S7 (A to D)). The role of the acid dopants is to enhance the hydrogen bonding dynamics within the solvent molecular network, while increasing the ionic strength of the DES and improving diffusion. Moreover, the anions act as doping agents in the polymer film, thus improving the charge transfer rate. The influence of the addition of different acid dopants has been noted in previous studies [12].

Cyclic voltammograms obtained during electropolymerization showed better peak definitions and higher currents when the dopants HClO_4_ and H_2_SO_4_ were used for both polymers, as seen in Figs. S6C and S6D, and Figs. S7C and S7D, respectively. When tested in 0.1 M BR buffer pH 3 (Fig. S6E for PBCG and Fig. S7E for PBCP), the polymer films prepared with the addition of HClO_4_ presented the highest anodic peak current density *j*_p_(II_a_) = 35.3 ± 0.03 µA cm^−2^ than H_2_SO_4_ *j*_p_(II_a_) = 14.6 ± 0.04 µA cm^−2^, HCl *j*_p_(II_a_) = 23.0 ± 0.03 µA cm^−2^, and HNO_3_ *j*_p_(II_a_) = 18.4 ± 0.05 µA cm^−2^ for PBCG. The same was observed for PBCP with the highest anodic peak current density for HClO_4_ *j*_p_(II_a_) = 13.8 ± 0.01 µA cm^−2^ higher than H_2_SO_4_ *j*_p_(II_a_) = 10.1 ± 0.03 µA cm^−2^, HCl *j*_p_(II_a_) = 10.9 ± 0.04 µA cm^−2^, and HNO_3_ *j*_p_(II_a_) = 8.8 ± 0.01 µA cm^−2^. Thus, HClO_4_ was chosen as the acid dopant for further studies.

### Electrochemical impedance spectroscopy

**Fig. 2:**
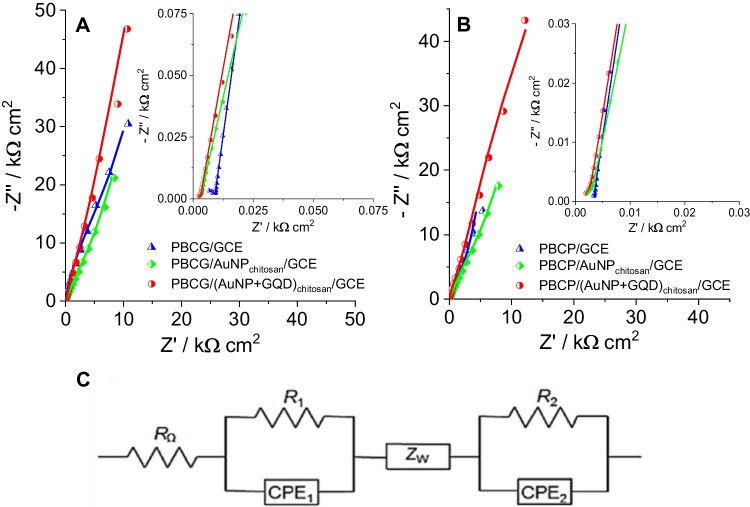
**A** Complex plane impedance spectra of PBCG with different modifications PBCG/GCE, PBCG/AuNP_chitosan_/GCE and PBCG/(AuNP+GQD)_chitosan_/GCE. **B** Complex plane impedance spectra of PBCP with different modifications PBCP/GCE, PBCP/AuNP_chitosan_/GCE and PBCP/(AuNP+GQD)_chitosan_/GCE synthesized in tDES with 1 M HClO_4_ as acid dopant at 50 mV s^−1^. Measurements were made in 0.1 M BR buffer solution (pH 3.0). **C** Full electrical equivalent circuit used to fit the spectra recorded at 0.3 V vs. Ag/AgCl shown in Fig. 4A and B

EIS was used to evaluate the influence of different modifications on the electropolymerization of PBCG and PBCP (see Fig. 2). The applied potential chosen was 0.3 V vs. Ag/AgCl, since it is close to and between the potentials at which the PBCG and PBCP current peaks are observed in 0.1 M BR buffer solution, pH 3, and to the open-circuit potential of 0.25 V. The equivalent circuit used to model the spectra is presented in Fig. 2C, and has been used in previous work with these electroactive redox polymer-modified electrodes, e.g. [3]. The equivalent circuit comprises a solution resistance *R*_Ω_, followed by a parallel combination of *R*_1_ and CPE_1_, which models the interfacial region between the GCE and the modifying films, a Warburg element (*Z*_w_), which defines the diffusional process in the modifying film and a second parallel combination of *R*_2_ and CPE_2_ that characterizes the interface between the modifying films and the solution interface. The Warburg element is defined by *Z*_W_ = *R*_W_cth[([(τiω)^α^](τiω)^−α^, where *α* ≤ 0.5, *R*_W_ is the diffusional resistance, and τ is the diffusion time constant. The constant phase element, CPE, is expressed by CPE = 1/(*C*i*ω*)^*α*^, where *C* is the capacitance, *ω* is the radial frequency, and *α* is the CPE exponent, varying between 0.5 ≤ *α* ≤ 1, where the value of 0.5 corresponds to a porous electrode and the value of 1 to a uniform and smooth surface [24]. The values of the circuit parameters obtained by fitting the spectra corresponding to different modifications for PBCG and PBCP to the equivalent electrical circuit are displayed in Tables S1 and S2, respectively. The part of the spectra at high frequency and its fitting is more visible in the inserts of Fig. 2A and B.

In Fig. 2A, the spectra of PBCG polymer film-coated bare GCE electrodes, AuNP_chitosan_/GCE and (AuNP+GQD)_chitosan_/GCE, are shown. The *α*_1_ (CPE_1_ exponent) values for all modifications were close to 1, indicating a uniform surface at the interface between the GCE substrate and the modifier film. A smaller impedance was observed for PBCG polymer films on AuNP_chitosan_/GCE and (AuNP+GQD)_chitosan_/GCE compared to bare GCE, demonstrating greater charge separation and lower charge transfer resistance (see insert of Fig. 2A). The values of *Z*_w_ were very similar for PBCG prepared in AuNP_chitosan_/GCE and (AuNP+GQD)_chitosan_/GCE, respectively, which indicates that the PBCG/AuNP_chitosan_ and PBCG/(AuNP+GQD)_chitosan_ polymer films have similar thicknesses, and the *α*_ZW_ value is close to 0.5 for all scan rates, indicating uniform diffusion over the area of the electrode. The *α*_2_ values approach 1, indicating a highly uniform polymer film modifier-solution interface at the nanoscale.

In Fig. 2B, the spectra of PBCP polymer formation at different modifications are shown: bare GCE, AuNP_chitosan_/GCE, and (AuNP+GQD)_chitosan_/GCE. The value of *R*_1_ decreases, and that of CPE_1_ increases. This behavior was observed when the electrode was modified with AuNP_chitosan_ and (AuNP+GQD)_chitosan_, indicating a greater charge separation and easy electron transfer. It can be explained by the synergistic effects established between the AuNP_chitosan_, (AuNP+GQD)_chitosan_, and the PBCP polymer. A higher value of *α*_1_ was observed for PBCP/AuNP_chitosan_/GCE and PBCP/(AuNP+GQD)_chitosan_/GCE, suggesting that a more uniform and smoother polymer film was obtained when AuNP and GQD were incorporated on the GCE surface, as shown in Table S2. The values of *Z*_W_ were very similar, which indicates that the AuNP_chitosan_/polymer and (AuNP+GQD)_chitosan_/polymer films have similar thicknesses. The values of *α*_ZW_ were approximately 0.45, indicating a uniform, close to smooth, interface.

### Characterization of PBCG/(AuNP+GQD)_chitosan_/GCE and PBCP/(AuNP+GQD)_chitosan_/GCE

#### Cyclic voltammetry

**Fig. 3:**
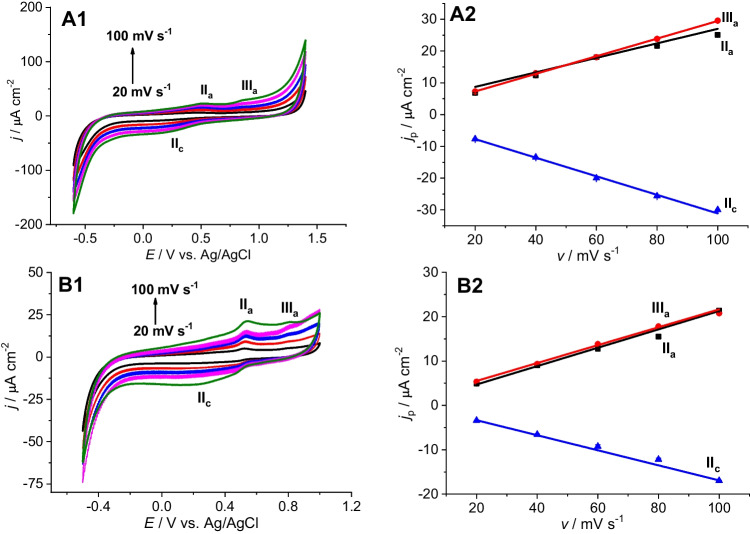
(A1 and B1) Cyclic voltammograms of PBCG/(AuNP+GQD)_chitosan_/GCE and PBCP/(AuNP+GQD)_chitosan_/GCE, respectively, in 0.1 M BR solution (pH 3.0) for scan rates 20 to 100 mV s^−1^. (A2 and B2) Plots of peak current II_a_/II_c_ vs. scan rate; error bars (3 modified electrodes) are too small to be visible. All CVs start at the negative potential limit in the positive direction

The PBCG/(AuNP+GQD)_chitosan_/GCE and PBCP/(AuNP+GQD)_chitosan_/GCE modified electrodes prepared in tDES, with the addition of HClO_4_ acid dopant, were characterized in 0.1 M BR solution (pH 3.0) by CV in the scan rate range 20–100 mV s^−1^ (Fig. 3A1 and B1). The voltammograms show a linear dependence of the anodic and cathodic peak current densities *j*_pa_ and *j*_pc_ on the scan rate for redox couples II_a_/II_c_ for PBCG and PBCP polymer (Fig. 3A2 and B2), respectively, indicating that the nature of the redox process is surface-confined within the modifier layer. The corresponding equations (*n* = 3) for PBCG are: *j*_pa2_ (µA cm^−2^) = (4.157 ± 1.749) + (0.228 ± 0.028) *v* (*R*^2^ = 0.941); *j*_pa3_ (µA cm^−2^) = (1.739 ± 0.19) + (0.277 ± 0.003) *v* (*R*^2^ = 0.999) and *j*_pc2_ (µA cm^−2^) = (−1.854 ± 0.378) − (0.293 ± 0.007) *v* (*R*^2^ = 0.997). For PBCP, the equations (*n* = 3) are: *j*_pa2_ (µA cm^−2^) = (0.564 ± 0.867) + (0.207 ± 0.01) *v* (*R*^2^ = 0.991); *j*_pa3_ (µA cm^−2^) = (1.478 ± 0.579) + (0.202 ± 0.008) *v* (*R*^2^ = 0.994) and *j*_pc2_ (µA cm^−2^) = (0.06 ± 0.24) − (0.17 ± 0.003) *v* (*R*^2^ = 0.999).

#### Scanning electron microscopy

**Fig. 4:**
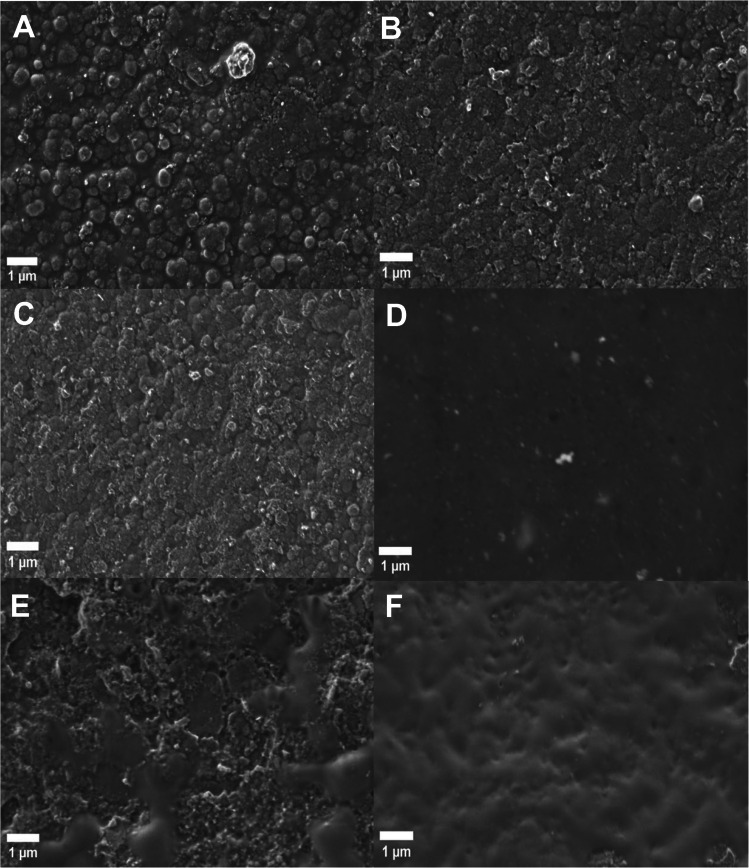
SEM micrographs: **A** carbon film electrode (CFE), **B** PBCG/CFE, **C** PBCP/CFE, **D** (AuNP + GQD)_chitosan_/CFE, **E** PBCG/(AuNP + GQD)_chitosan_/CFE, and **F** PBCP/(AuNP + GQD)_chitosan_/CFE

The PBCG and PBCP polymer film morphology was characterized by SEM (see Fig. 4). The surface morphology of the unmodified CFE is presented in Fig. 4A, revealing an irregular surface. Figure 4B and C show the images of the polymer film on CFE, PBCG/CFE, and PBCP/CFE, respectively, which have an irregular surface with some smoothing of the structure compared to the bare CFE.

Figure 4D shows that modification to give (AuNP+GQD)_chitosan_/CFE is characterized by light-colored spherical spots attributed to the metal nanoparticles; the different spot sizes of (AuNP+GQD)_chitosan_ on the electrode surface are attributed to the presence of GQD, which could contribute to the aggregation of the AuNP. Figure 4E and F show images of PBCG/(AuNP+GQD)_chitosan_/CFE and PBCP/(AuNP+GQD)_chitosan_/CFE, respectively, showing that the polymer films cover the (AuNP+GQD)_chitosan_. Compared to the unmodified CFE, more homogeneous and smoother surfaces are obtained on PBCG/(AuNP+GQD)_chitosan_/CFE and PBCP/(AuNP+GQD)_chitosan_/CFE, but the morphology in Figure 4F (PBCP) is more uniform than in Figure 4E (PBCG) that shows a surface partly covered by platelets. This could be due to the polymer film structure being influenced by the differences in detailed monomer structure (some bromines vs. methyl group substituents on the aromatic rings, Scheme S1) altering size and polarity, thence affecting polymer film growth.

### GOx/PBCG/(AuNP+GQD)_chitosan_/GCE and GOx/PBCP/(AuNP+GQD)_chitosan_/GCE as glucose biosensors

#### Cyclic voltammetric behavior of the biosensors

**Fig. 5:**
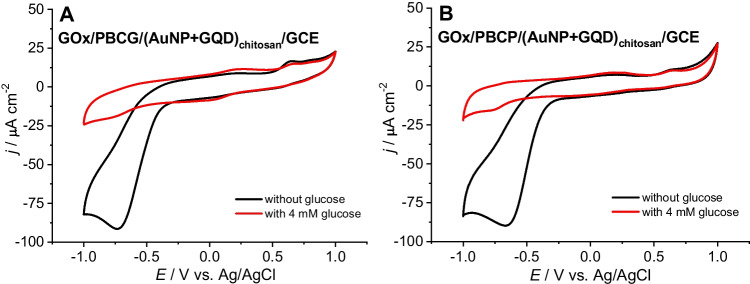
CVs with and without glucose in 0.1 M NaPBS (pH 7.0) in the potential range −1.0 to +1.0 V vs. Ag/AgCl at **A** GOx/PBCG/(AuNP+GQD)_chitosan_/GCE and **B** GOx/PBCP/(AuNP+GQD)_chitosan_/GCE at scan rate 50 mV s−1, starting at the negative potential limit in the positive direction

Glucose oxidase enzyme modified PBCG/(AuNP+GQD)_chitosan_/GCE and PBCP/(AuNP+GQD)_chitosan_/GCE were prepared by glutaraldehyde cross-linking immobilization, following the procedure described by Ghica and Brett [17]. The redox behavior of glucose at the biosensors was then examined by cyclic voltammetry in the potential range −1.0 V to +1.0 V vs. Ag/AgCl in 0.1 M NaPBS pH 7.0. The CVs in Fig. 5A and B, recorded without and with glucose, show the catalytic activity associated with the GOx enzyme interaction with the redox mediator polymer films and nanomaterials.

The reduction response below −0.5 V vs. Ag/AgCl, respectively, without glucose (black line) for both sensors can be attributed to oxygen reduction. On addition of glucose (red line), oxygen is consumed and oxidative regeneration of the FAD (flavin adenine dinucleotide) cofactor of GOx (reduced by reaction with glucose) at PBCG or PBCP occurs, leading to an anodic change in current. This behavior was also observed in Ghica and Brett [17] when employing poly(neutral red) as redox mediator in glucose biosensors.

#### Amperometric determination of glucose

**Fig. 6:**
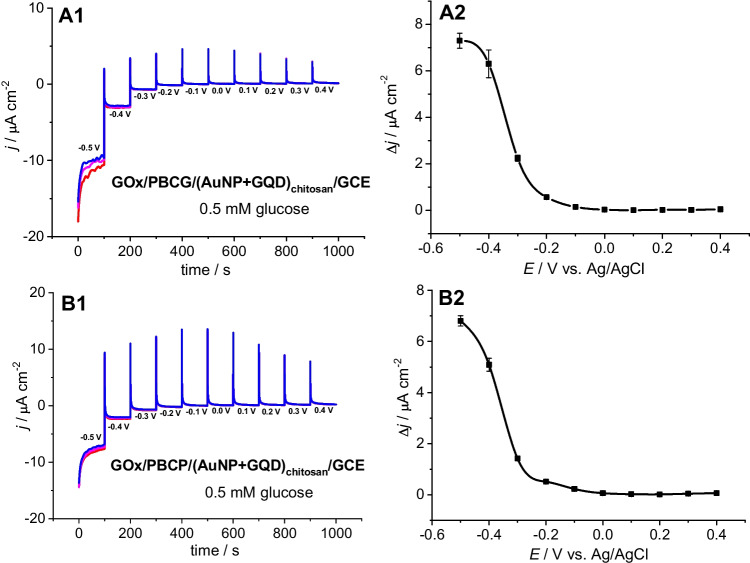
Effect of applied potential on response to 0.5 mM glucose at GOx/PBCG/(AuNP+GQD)_chitosan_/GCE and GOx/PBCP/(AuNP+GQD)_chitosan_/GCE, respectively. (A1 and B1) Current responses. (A2 and B2) Differences in current with glucose (data from A1 and B1); error bars from 3 replicas are too small to be visible for potentials ≥ 0.3 V

After optimizing the conditions for preparing GOx/PBCG/(AuNP+GQD)_chitosan_/GCE and GOx/PBCP/(AuNP+GQD)_chitosan_/GCE sensors, they were applied to glucose measurement by fixed-potential amperometry. Various potentials were tested between −0.50 and +0.40 V vs. Ag/AgCl in 0.1 M NaPBS pH 7.0 (see Fig. 6).

The potential −0.20 V vs. Ag/AgCl was chosen, even though the sensitivity was lower, based on the experimentally determined better reproducibility of recording the amperograms to construct the calibration curves, for both GOx/PBCG/(AuNP+GQD)_chitosan_/GCE and GOx/PBCP/(AuNP+GQD)_chitosan_/GCE.

**Fig. 7:**
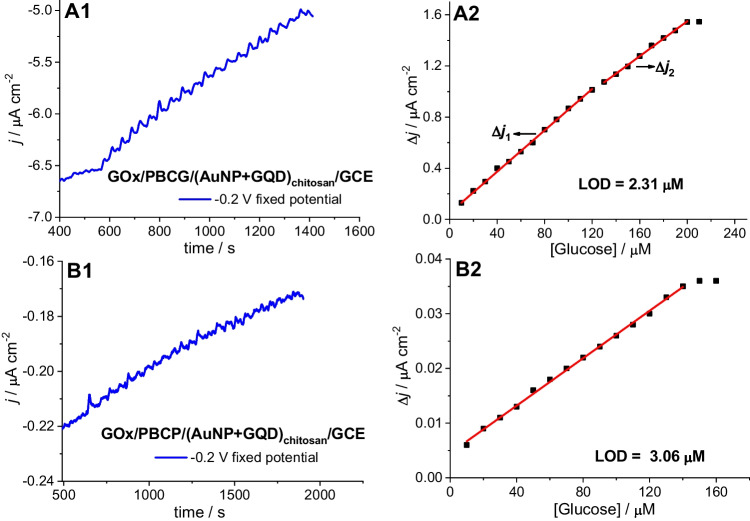
(A1 and B1) Amperometric response at GOx/PBCG/(AuNP+GQD)_chitosan_/GCE and GOx/PBCP/(AuNP+GQD)_chitosan_/GCE, respectively for successive additions of glucose at applied potential −0.2 V vs. Ag/AgCl in 0.1 M NaPBS (pH 7.0) in the concentration range 10–200 µM for GOx/PBCG/(AuNP+GQD)_chitosan_/GCE and 10–140 µM GOx/PBCP/(AuNP+GQD)_chitosan_/GCE. (A2 and B2) Corresponding calibration plot; error bars are too small to be visible

Figure 7A1 and B1 show amperograms for consecutive additions of 20 µL of a 1 mM glucose standard solution in 0.1 M NaPBS (pH 7.0), after stabilization of the current for 500 s, corresponding to final concentrations of 10 to 200 µM for GOx/PBCG/(AuNP+GQD)_chitosan_/GCE and 10 to 160 µM for GOx/PBCP/(AuNP+GQD)_chitosan_/GCE.

The limit of detection (LOD) and quantification (LOQ) were calculated using the Eqs. 3*s*_B_/*b* and 10*s*_B_/*b*, respectively, *s*_B_ being the standard deviation of the intercept and *b* the slope of the calibration plot. The calculated LOD and LOQ values were 2.31 µM and 7.69 µM for GOx/PBCG/(AuNP+GQD)_chitosan_/GCE, and 3.06 µM and 10.20 µM for GOx/PBCP/(AuNP+GQD)_chitosan_/GCE, respectively. Comparison between the two biosensors shows that the analytical parameters for glucose determination at GOx/PBCG/(AuNP+GQD)_chitosan_/GCE were a little better, with lower LOD and LOQ and a longer linear range, than at GOx/PBCP/(AuNP+GQD)_chitosan_/GCE.

The calibration plot in Fig. 7A2 (two segments) and Fig. 7B2 for both sensors is constructed with data from the amperograms in Fig. 7A1 and B1, respectively, and can be described by the linear regression equation (*n* = 3): ∆*j*_1_ (µA cm^−2^) = (0.0496 ± 0.0066) + (0.0081 ± 8.0279 × 10^–5^) [glucose] (µM) (*R*^2^ = 0.999) in the range (10 to 120 µM) with a sensitivity of (0.0081 ± 8. 0279 × 10^–5^ µA µM^−1^ cm^−2^) and ∆*j*_2_ (µA cm^−2^) = (0.1825 ± 0.0229) + (0.0069 ± 1.3735 × 10^–4^) [glucose] (µM) (*R*^2^ = 0.997) in the range (130 to 200 µM) with a sensitivity of (0.0069 ± 1.3235 × 10^–4^ µA µM^−1^ cm^−2^) for GOx/PBCG/(AuNP+GQD)_chitosan_/GCE. For GOx/PBCP/(AuNP+GQD)_chitosan_/GCE, the linear equation (*n* = 3) was: ∆*j* (µA cm^−2^) = (0.0045 ± 2.2115 × 10^–4^) + (2.1692 × 10^–4^ ± 2.5974 × 10^–6^) [glucose] (µM) (*R*^2^ = 0.998) in the range (10 to 140 µM) with a sensitivity of (0.0333 ± 0.0009 µA µM^−1^ cm^−2^).

Analytical parameters of the GOx/PBCG/(AuNP+GQD)_chitosan_/GCE and GOx/PBCP/(AuNP+GQD)_chitosan_/GCE sensors for determining glucose, and of similar sensors and biosensors available in the literature, are presented in Table 1. The new biosensors, GOx/PBCG/(AuNP+GQD)_chitosan_/GCE and GOx/PBCP/(AuNP+GQD)_chitosan_/GCE, have analytical parameters comparable to the other sensors in the determination of glucose, namely the limit of detection (LOD) and linear range, demonstrating that the GOx/PBCG/(AuNP+GQD)_chitosan_/GCE and GOx/PBCP/(AuNP+GQD)_chitosan_/GCE prepared in tDES are promising platforms for electrochemical sensing of glucose. Electrode modification is simple using AuNP and GQD nanomaterial dispersions that are easy to prepare, and are cheap and eco-friendly.

**Table 1:** Determination of glucose at different modified electrode sensors and glucose oxidase biosensors in the literature

Sensor architecture	Enzyme	Technique	LOD ([µM)	Linear range (µM)	Ref
PBCB/GOx/GCE	GOx	Amp	31	50–1300	[17]
GNs/NB_poly_/GCE	GOx	CV	2.1	200–2000	[6]
GOx-HRP-pTB/RGO/GCE	GOx-HRP	Amp	50	80–1000	[25]
Cu/Co-bilayered NWs/copper tape	GOx	DPV	0.05	0.3–2.6	[26]
Nafion/CuO nanofibers/GCE	-	CV	0.20	100–10850	[27]
Nafion-GOx/GO/AZO/Ag	GOx	Potentio	1890	2000–10000	[28]
GOx/Nafion/[Demim]Br/PANI-TNTs/GCE	GOx	DPV	0.5	10–2500	[29]
NiCo_2_O_4_@PANI/GCE	-	Amp	0.38	15–4740	[30]
HNTs/AgNPs/GCE	GOx	CV	200	200–6000	[31]
Nafion/GOx/Nafion platinized paper	GOx	Potentio	100	100–10000	[23]
Co_3_O_4_/GR microspheres/GCE	-	Amp	0.04	20–800	[32]
Nafion/GOx/MnO_2_ NWs/GCE	GOx	Amp	1.8	5–2000	[33]
CCAu/Nafion/GCE	-	Amp	1.7	1–45	[34]
Au/CuO nanocomposites/GCE	-	Amp	10	5–3550	[35]
GOx/PBCG/(AuNP+GQD)_chitosan_/GCE	GOx	Amp	2.3	10–200	This work
GOx/PBCP/(AuNP+GQD)_chitosan_/GCE	GOx	Amp	3.1	10–140	This work

*PBCB*, poly(brilliant cresyl blue); *GOx*, glucose oxidase; *GCE*, glassy carbon electrode; *Amp.*, amperometry; *GNs*, graphene; *NB*_*poly*_, Nile blue; *CV*, cyclic voltammetry; *HRP*, horseradish peroxidase; *pTB*, poly(toluidine blue); *RGO*, reduced graphene oxide; *Cu/Co-bilayered NWs*, copper/cobalt bilayered nanowires; *DPV*, differential pulse voltammetry; *CuO*, copper(II) oxide; *GO/AZO*, graphene oxide-aluminum-doped zinc oxide; *Potentio.*, potentiometric; *[Demim]Br*, brominated 1-decyl-3-methyl imidazole; *PANI*, polyaniline; *TNTs*, TiO_2_ nanotubes; *HNTs*, halloysite nanotubes; *AgNP*, silver nanoparticles; *GR*, graphene; *NWs*, nanowires; *CCAu*, ternary flower-like Cu_2_O-Cu-Au

#### Repeatability, reproducibility, and stability of the biosensors

The repeatability of the GOx/PBCG/(AuNP+GQD)_chitosan_/GCE and GOx/PBCP/(AuNP+GQD)_chitosan_/GCE sensors in determining glucose was evaluated by amperometry. Ten consecutive measurements of four different concentrations were made (25, 75, 150, and 250 µM) at −0.20 V vs. Ag/AgCl and the RSD% were 1.03 ± 0.12%, 0.39 ± 0.03%, 0.23 ± 0.01%, and 0.13 ± 0.02% (*n* = 10) for GOx/PBCG/(AuNP+GQD)_chitosan_/GCE and the RSD% were 3.23 ± 0.12%, 3.23 ± 0.15%, 3.85 ± 0.21%, and 3.85 ± 0.23% (*n* = 10) for GOx/PBCP/(AuNP+GQD)_chitosan_/GCE, respectively.

Four GOx/PBCG/(AuNP+GQD)_chitosan_/GCE and GOx/PBCP/(AuNP+GQD)_chitosan_/GCE were prepared independently under the same conditions to evaluate reproducibility. Using 1 mM glucose, calibration plots led to RSD% in the sensitivity of 2.81 ± 0.02% for GOx/PBCG/(AuNP+GQD)_chitosan_/GCE and 2.67 ± 0.13% for GOx/PBCP/(AuNP+GQD)_chitosan_/GCE.

The stability of GOx/PBCG/(AuNP+GQD)_chitosan_/GCE and GOx/PBCP/(AuNP+GQD)_chitosan_/GCE was assessed during a period of a month and a half (42 days), making measurements once a week. The modified electrode was stored in 0.1 M NaPBS (pH 7.0) in the refrigerator at 4 °C. The response decreased by 6.0 ± 0.4% for GOx/PBCG/(AuNP+GQD)_chitosan_/GCE and 8.0 ± 0.1% for GOx/PBCP/(AuNP+GQD)_chitosan_/GCE, respectively, compared to the initial response. The high stability is attributed to the affinity between the analyte and the polymer films prepared in tDES.

Analyzing these data, the GOx/PBCG/(AuNP+GQD)_chitosan_/GCE was slightly better in terms of repeatability, and in stability in response to glucose, than GOx/PBCP/(AuNP+GQD)_chitosan_/GCE. Reproducibility was slightly better for the PBCP-based biosensor.

#### Selectivity and interferences

The selectivity of the GO_x_/PBCG/(AuNP+GQD)_chitosan_/GCE and GOx/PBCP/(AuNP+GQD)_chitosan_/GCE biosensors was evaluated by determining glucose by fixed potential amperometry at −0.20 V vs. Ag/AgCl in the presence of possible interferents in the proportion 1:1 (glucose:interferents), i.e., CC, Fru, UA, AA, CA, TTA, APAP, and LA, Figs. S8A and S8B, for applications in wine, urine, and blood samples.

Injections of 40 µL of a 1 mM glucose standard were made into the cell containing 2 mL of 0.1 M NaPBS (pH 7.0), corresponding to 20 µM in the cell, before and after the addition of the interferents. The added interferents (40 µL of each) were all prepared with a concentration of 1 mM in 0.1 M NaPBS (pH 7.0), becoming 20 µM after injection into the cell corresponding to a 1:1 molar ratio of glucose to interferents. The interferents CC, Fru, UA, CA, TTA, APAP, and LA with these concentrations did not show a response at the applied potential used and did not affect the detection of glucose.

For AA, CA, TTA, and LA, which could exist at higher concentrations in wine samples, complementary analyses at higher concentrations were done to mimic such situations, and interferences up to 40% could be obtained in the least favorable cases. However, such possible interferences should play a reduced role in the determination of glucose using the standard addition method.

#### Analysis of wine samples

The GOx/PBCG/(AuNP+GQD)_chitosan_/GCE and GOx/PBCP/(AuNP+GQD)_chitosan_/GCE biosensors were used to analyze glucose in red and white wines (see Fig. S9). Sample preparation is described in the “Experimental” section. Calibration plots were constructed using fixed potential amperometry at −0.2 V vs. Ag/AgCl and the standard addition method to minimize the influence of the sample matrix. The plots follow the linear regression equations (*n* = 3): ∆*j* (µA cm^−2^) = (0.041 ± 0.028) + (0.0032 ± 0.001) [glucose] (µM) (*R*^2^ = 0.998) for the red wine sample and ∆*j* (µA cm^−2^) = (0.127 ± 0.063) + (0.0039 ± 0.001) [glucose] (µM) (*R*^2^ = 0.996) for the white wine sample for the GOx/PBCG/(AuNP+GQD)_chitosan_/GCE sensor. The plots for GOx/PBCP/(AuNP+GQD)_chitosan_/GCE sensor were ∆*j* (µA cm^−2^) = (0.0033 ± 0.0002) + (1.723 × 10^–4^ ± 0.00003) [glucose] (µM) (*R*^2^ = 0.998) for the red wine sample and ∆*j* (µA cm^−2^) = (0.0026 ± 0.0002) + (1.336 × 10^–4^ ± 0.00003) [glucose] (µM) (*R*^2^ = 0.995) for the white wine sample.

For all samples, the correlation coefficients are above *R*^2^ = 0.99. The concentrations found were very close to the expected concentrations, demonstrating efficiency and reliability of the electrochemical method with good recoveries (see Table 2). RSD values were 5.0 ± 0.4% and 4.9 ± 0.5% for the GOx/PBCG/(AuNP+GQD)_chitosan_/GCE sensor, and 4.6 ± 0.6% and 2.9 ± 0.6% for the GOx/PBCP/(AuNP+GQD)_chitosan_/GCE sensor. The sample dilution made it possible to determine glucose without interference from excipients with good repeatability and reproducibility.

**Table 2:** Determination of glucose in wine samples (*n* = 3) obtained at GOx/PBCG/(AuNP+GQD)_chitosan_/GCE and GOx/PBCP/(AuNP+GQD)_chitosan_/GCE; polymer films prepared in tDES

Wine sample	Expected concentration (µM)	Found concentration (µM)	Recovery (%)
GOx/PBCG/(AuNP+GQD)_chitosan_/GCE			
Red wine	15.0	13.7 ± 0.7	91.3 ± 0.6
White wine	30.0	32.8 ± 1.7	109.3 ± 0.8
GOx/PBCP/(AuNP+GQD)_chitosan_/GCE			
Red wine	20.0	20.2 ± 0.9	101.1 ± 0.5
White wine	20.0	19.1 ± 0.6	95.5 ± 0.7

## Conclusions

The redox polymer films PBCG and PBCP were successfully deposited by potential cycling electropolymerization on (AuNP+GQD)_chitosan_/GCE in tDES. GQD were synthesized using citric acid as a carbon source, were dispersed in chitosan solution, and were then mixed with a chitosan dispersion of AuNP previously stabilized in citrate buffer. The electrochemical properties of the PBCG/(AuNP+GQD)_chitosan_/GCE and PBCP/(AuNP+GQD)_chitosan_/GCE sensors were investigated and compared by CV and EIS, given the similarities in monomer structure; the morphology was characterized by SEM. The slightly different monomer structures, with two bromine substituents in the BCG ring being replaced by methyl in BCP, led to small differences in the polymer film electrochemical characteristics and surface morphology. Glucose oxidase enzyme was immobilized on the modified electrodes to make glucose biosensors. The analytical characteristics of the biosensors were evaluated by amperometry at fixed potential, and no significant interferences were observed. The GOx/PBCG/(AuNP+GQD)_chitosan_/GCE and GOx/PBCP/(AuNP+GQD)_chitosan_/GCE biosensors showed good repeatability, reproducibility, stability, and low detection limits (2.3 μM and 3.1 μM) for glucose, respectively, with slightly better analytical parameters for the PBCG-containing biosensor. Both biosensors are simple and easy to prepare and show the advantage of the combination of AuNP with GQD as (AuNP+GQD)_chitosan_ coated electrodes with polymer films prepared in the novel ChCl:AcA:EG ternary DES. The biosensors were successfully utilized to determine glucose in wine samples with good recoveries, which augurs well for future applications in foods and beverages.

## Supplementary Information

Below is the link to the electronic supplementary material.

Supplementary file1 (PDF 2.48 MB)

## Data Availability

Data will be made available on request.
