# Supplementary material for: Poly(bromocresol green) and poly(bromocresol purple) films by electropolymerization in ternary deep eutectic solvent on gold nanoparticle/graphene quantum dot modified electrodes for the biosensing of glucose
Source: Anal Bioanal Chem. 2026 May 4;418(14):4363–77. doi: 10.1007/s00216-026-06524-z (PMC13375703; doi:10.1007/s00216-026-06524-z)
Supplement: Supplementary file 1 — Supplementary file1 (PDF 2.48 MB) [file 216_2026_6524_MOESM1_ESM.pdf]

SUPPLEMENTARY INFORMATION

Poly(bromocresol green) and poly(bromocresol purple) films by electropolymerization in ternary deep eutectic solvent on gold nanoparticle/graphene quantum dot modified electrodes for the biosensing of glucose

Joseany M.S. Almeida[1] and Christopher M.A. Brett[1*]

[1] Department of Chemistry, CEMMPRE, ARISE, Faculty of Sciences and Technology, University of Coimbra, 3004-535 Coimbra, Portugal

* cbrett@ci.uc.pt

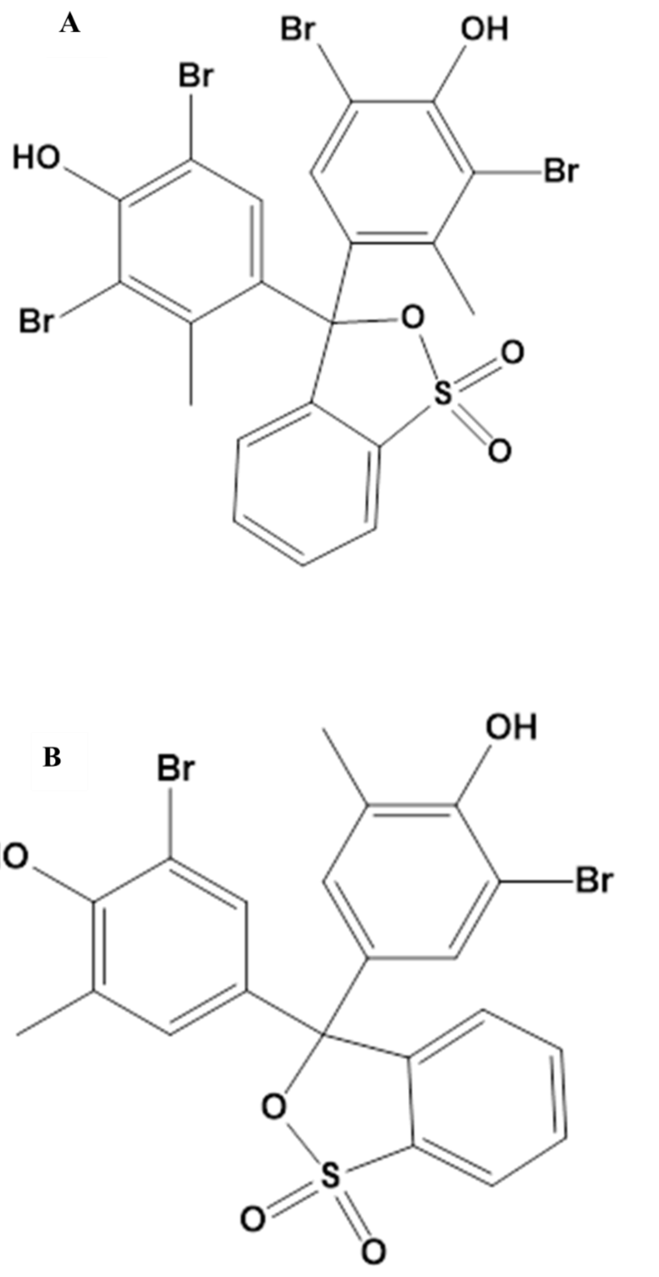

Scheme S1. Chemical structures of (A) bromocresol green (BCG) and (B) bromocresol purple (BCP).

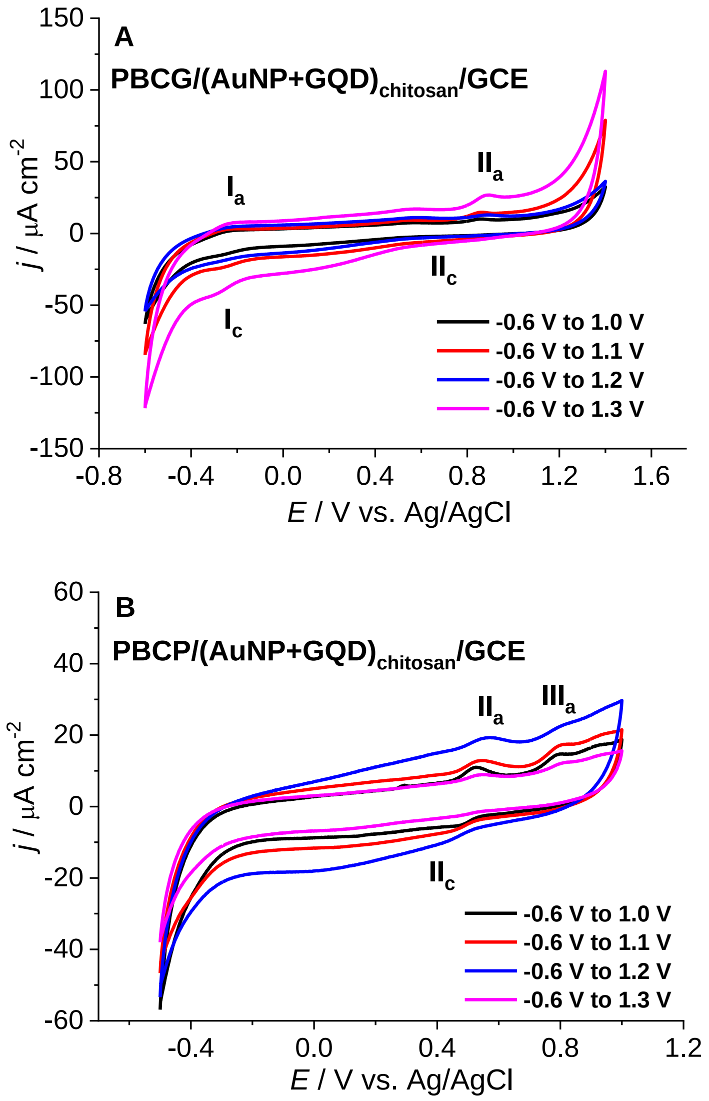

Fig. S1 CV curves in 0.1 M BR buffer (pH 3.0) for (A) PBCG/(AuNP+GQD)$_{chitosan}$/GCE and (B) PBCP/(AuNP+GQD)$_{chitosan}$/GCE after electropolymerization in ChCl:AcA:EG tDES at different positive potential limits (+1.0 V; +1.1 V; +1.2 V and +1.3 V vs. Ag/AgCl)

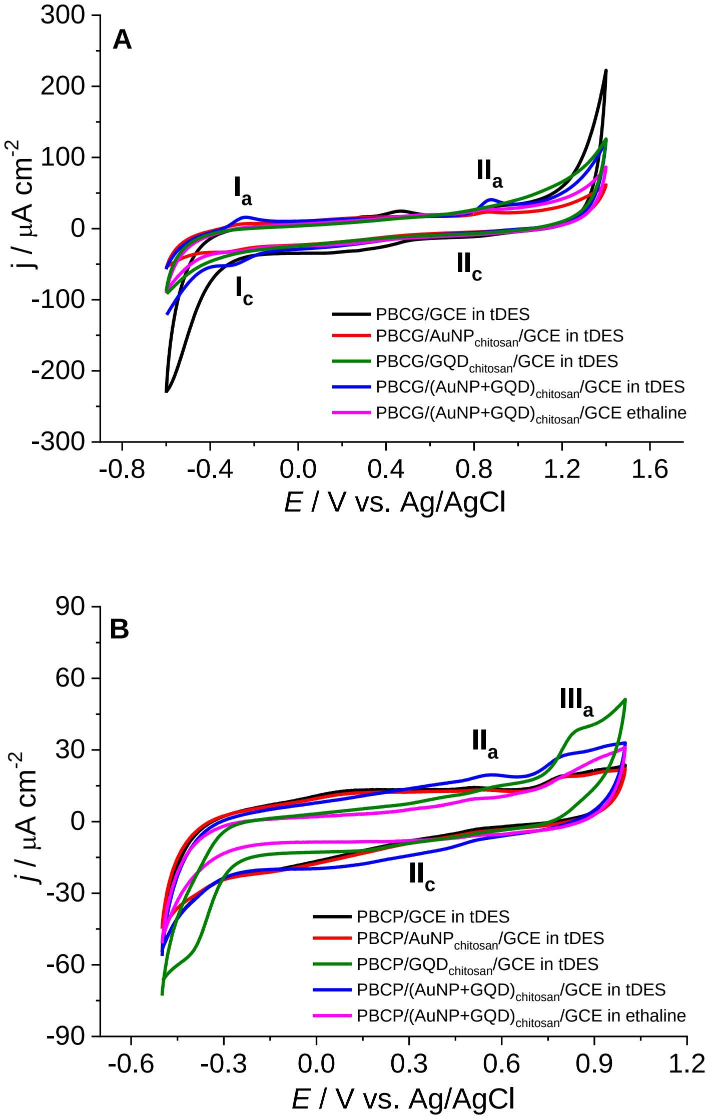

Fig. S2 CV curves in 0.1 M BR buffer (pH 3.0) of (A) PBCG/GCE, PBCG/$AuNP_{chitosan}$/GCE, PBCG/$GQD_{chitosan}$/GCE, PBCG/$(AuNP+GQD)_{chitosan}$/GCE after electropolymerization of BCG in ChCl:AcA:EG tDES and PBCG/$(AuNP+GQD)_{chitosan}$/GCE in ethaline. (B) PBCP/GCE, PBCP/$AuNP_{chitosan}$/GCE, PBCP/$GQD_{chitosan}$/GCE, PBCP/$(AuNP+GQD)_{chitosan}$/GCE after electropolymerization of BCP in ChCl:AcA:EG tDES and PBCP/$(AuNP+GQD)_{chitosan}$/GCE in ethaline.

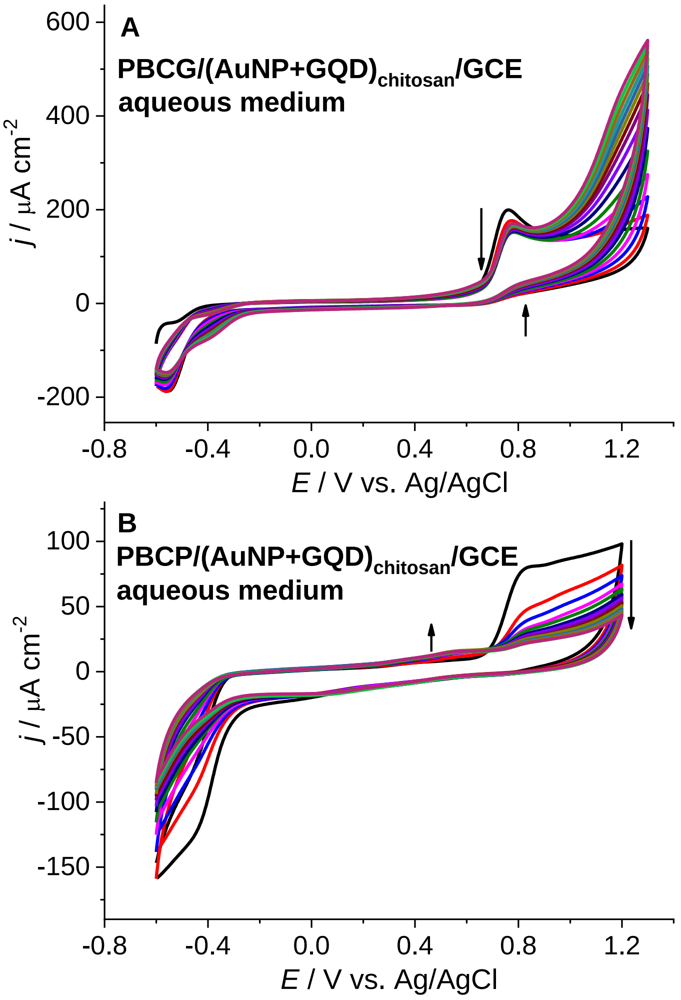

Fig. S3 Electropolymerization of 1mM (A) BCG (-0.6 V to +1.3 V vs. Ag/AgCl) and (B) BCP (-0.6 V to +1.2 V vs. Ag/AgCl) in aqueous medium (0.1 M NaOH plus 0.1M $HClO_4$) on $(AuNP+GQD)_{chitosan}$/GCE during 15 cycles at 100 mV s^{-1} scan rate.

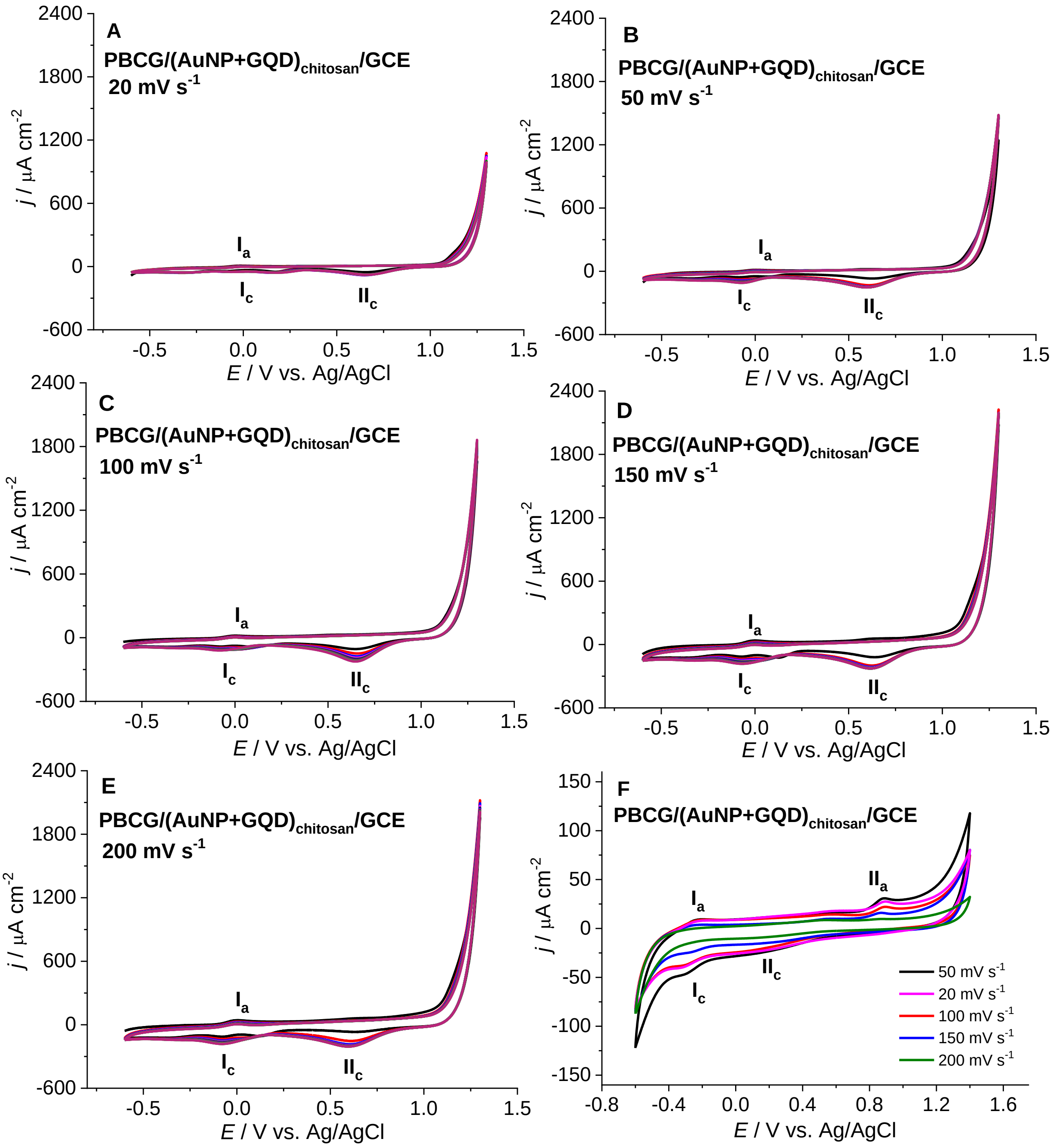

Fig. S4 Electropolymerization of 1 mM BCG in ChCl:AcA:EG tDES plus 1 M H_2SO_4 acid dopant on $(AuNP+GQD)_{chitosan}$/GCE potential range -0.6 V to +1.3 V vs. Ag/AgCl, 15 cycles, at scan rates (A) 20 mV s^{-1}, (B) 50 mV s^{-1}, (C) 100 mV s^{-1}, (D) 150 mV s^{-1} and (E) 200 mV s^{-1}. (F) CV curves of PBCG/$(AuNP+GQD)_{chitosan}$/GCE in 0.1 M BR buffer (pH 3.0).

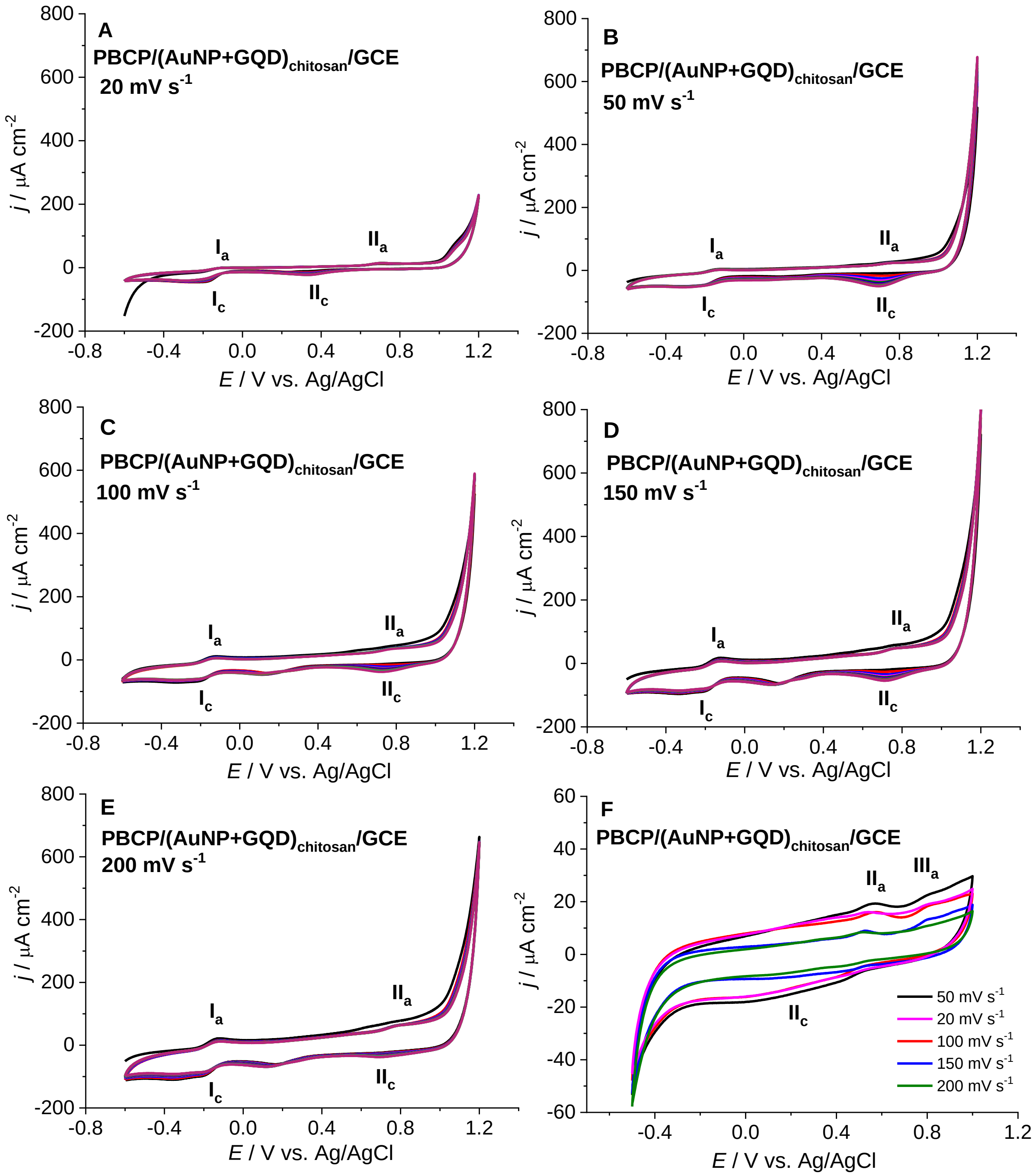

Fig. S5 Electropolymerization of 1 mM BCP in tDES (ChCl:AcA:EG 1:2:2 molar ratio) plus 1 M H_2SO_4 acid dopant on (AuNP+GQD)$_{chitosan}$/GCE potential range -0.6 V to +1.2 V vs. Ag/AgCl, 15 cycles, at scan rates (A) 20 mV s^{-1}, (B) 50 mV s^{-1}, (C) 100 mV s^{-1}, (D) 150 mV s^{-1} and (E) 200 mV s^{-1}. (F) CV curves of PBCP/(AuNP+GQD)$_{chitosan}$/GCE in 0.1 M BR buffer (pH 3.0).

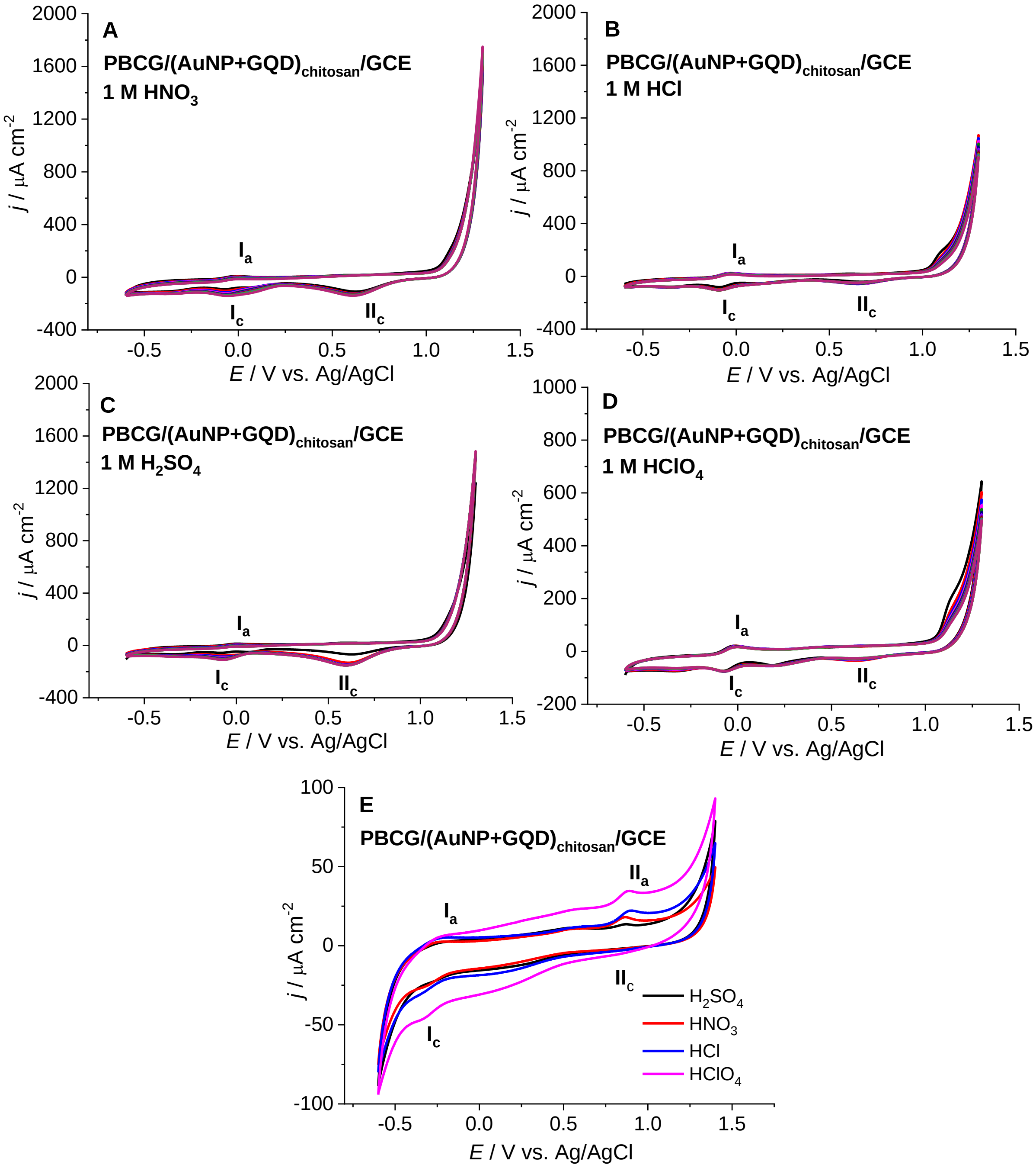

Fig. S6 Electropolymerization of 1mM BCG, potential range - 0.6 V to + 1.3 V vs. Ag/AgCl, scan rate 50 mV s^{-1}, 15 cycles, on (AuNP+GQD)$_{chitosan}$/GCE in tDES (ChCl:Ac:EG 1:2:2 molar ratio), with different acid dopants (1 M): (A) HNO_3, (B) HCl, (C) H_2SO_4 and (D) $HClO_4$. (E) CV curves of PBCG/(AuNP+GQD)$_{chitosan}$/GCE in 0.1 M BR buffer (pH 3.0).

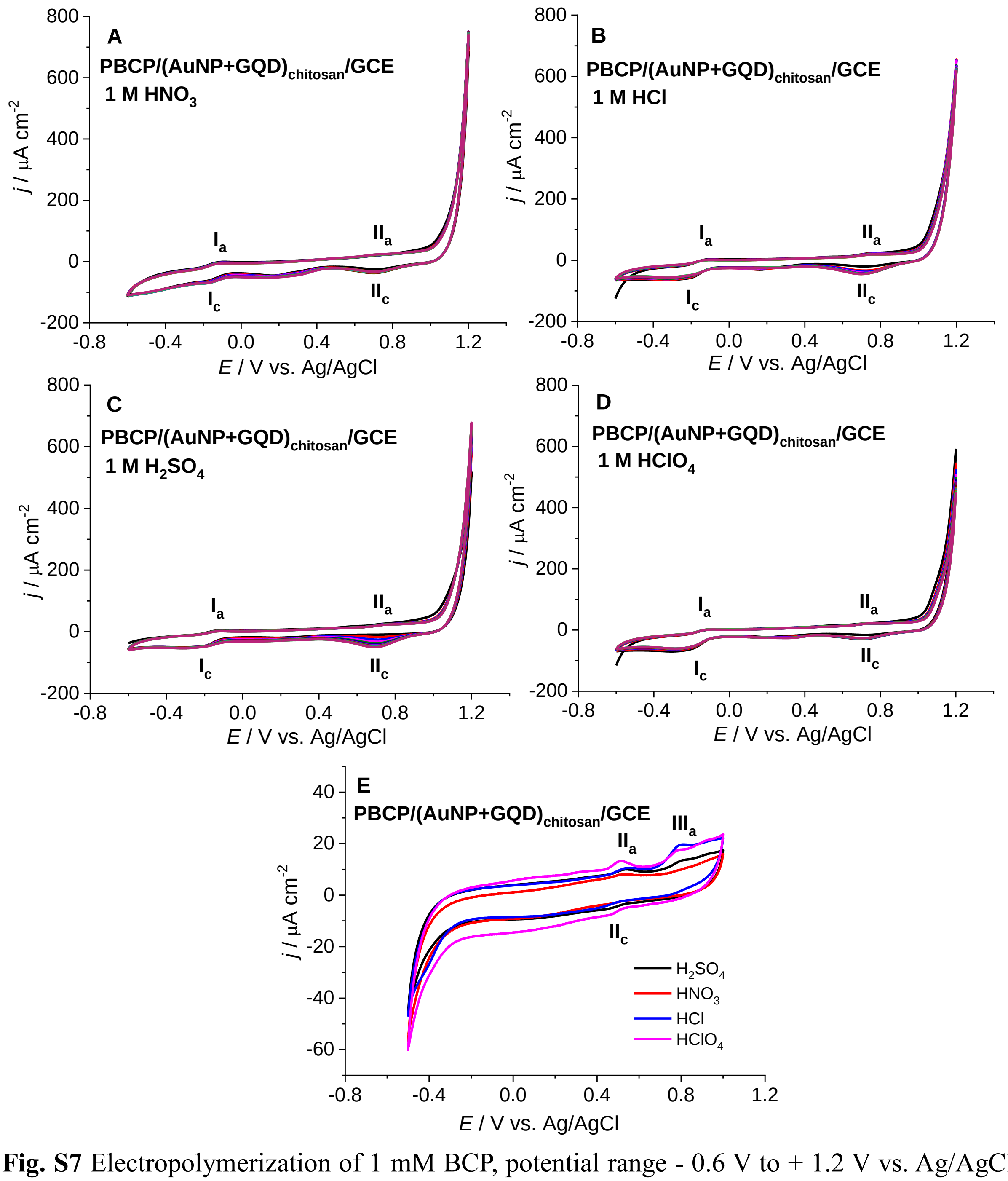

Fig. S7 Electropolymerization of 1 mM BCP, potential range - 0.6 V to + 1.2 V vs. Ag/AgCl, scan rate 50 mV s^{-1}, 15 cycles, on $(AuNP+GQD)_{chitosan}$/GCE in tDES (ChCl:Ac:EG 1:2:2 molar ratio), with different acid dopants. (A) HNO_3, (B) HCl, (C) H_2SO_4 and (D) $HClO_4$. (E) CV curves at PBCP/$(AuNP+GQD)_{chitosan}$/GCE in 0.1 M BR buffer (pH 3.0).

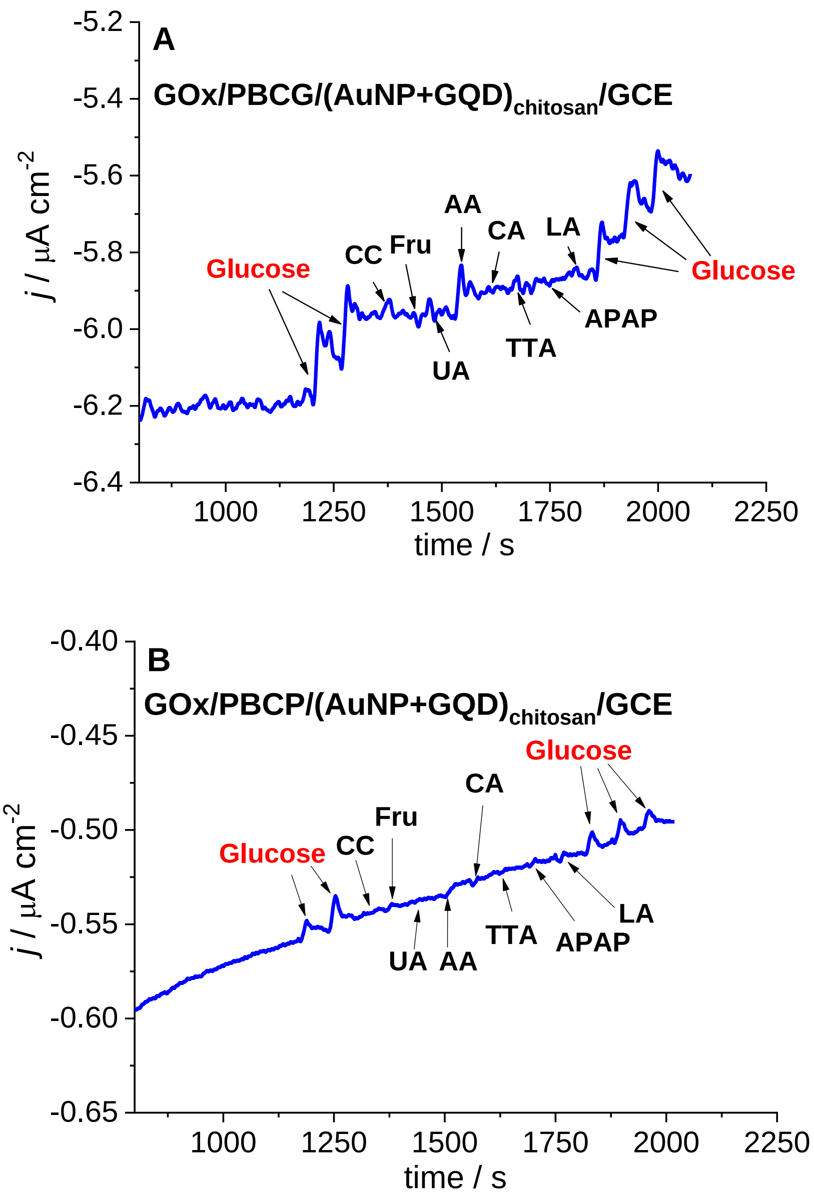

Fig. S8 Current response at - 0.2 V vs. Ag/AgCl in 0.1 M NaPBS (pH 7.0) at (A) GOx/PBCG/(AuNP+GQD)$_{chitosan}$/GCE and (B) GOx/PBCP/(AuNP+GQD)$_{chitosan}$/GCE toward 20 μM glucose, 20 μM CC, 20 μM Fruc, 20 μM UA, 20 μM AA, 20 μM CA, 20 μM TTA, 20 μM APAP and 20 μM LA, followed by two further injections of 1 mM glucose. Applied potential -0.2 V vs. Ag/ AgCl. See text for explanation of symbols.

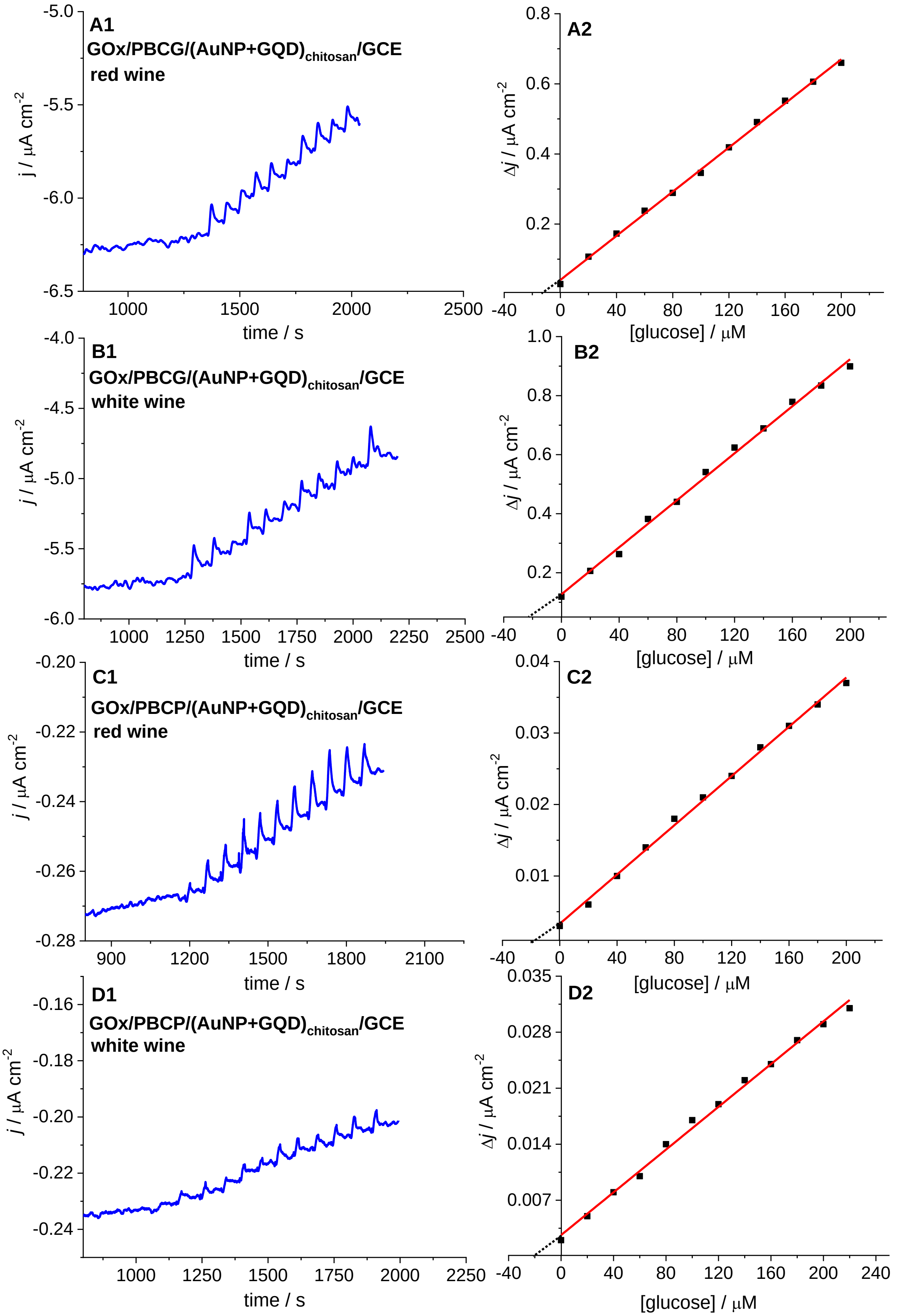

Fig. S9 Amperometric response in 0.1 M NaPBS (pH 7.0) using the standard addition method for quantification of glucose in (A1 and A2) red wine sample and (B1 and B2) white wine sample at a GOx/PBCG/(AuNP+GQD)$_{chitosan}$/GCE sensor and glucose in (C1 and C2) red wine sample and (D1 and D2) white wine sample at a GOx/PBCP/(AuNP+GQD)$_{chitosan}$/GCE sensor.

Table S1. Values of equivalent electrical circuit elements obtained by fitting the impedance spectra recorded at 0.3 V vs. Ag/AgCl in 0.1 M BR buffer solution (pH 3.0) (Fig. 2A) to the equivalent circuit in Figure 2C. The data relate to PBCG/GCE, PBCG/$AuNP_{chitosan}$/GCE and PBCG/$(AuNP+GQD)_{chitosan}$/GCE polymerization in ChCl:AcA:EG tDES at scan rate 50 mV s^{-1} with 1 M $HClO_4$ acid dopant.

GCE modifier	R_1 / Ω cm²	CPE_1 / µF cm^{-2} s$^{\alpha-1}$	α_1	Z_W / Ω cm²	α_{zw}	R_2 / Ω cm²	CPE_2 / mF cm^{-2}s$^{\alpha-1}$	α_2
PBCG	5.55 ± 0.93	0.229 ± 0.02	1 ± 0.05	2.42 ± 0.12	0.46 ± 0.06	9756 ± 1.5	0.072 ± 0.004	0.90 ± 0.13
PBCG/$AuNP_{chitosan}$	1.015 ± 0.35	0.175 ± 0.04	1 ± 0.04	0.232 ± 0.23	0.42 ± 0.03	1671 ± 2.3	0.082 ± 0.002	0.91 ± 0.21
PBCG/$(AuNP+GQD)_{chitosan}$	1.127 ± 0.76	0.421 ± 0.008	1 ± 0.03	0.381 ± 0.30	0.44 ± 0.05	647.4 ± 1.4	0.136 ± 0.001	0.95 ± 0.18

Table S2. Values of equivalent electrical circuit elements obtained by fitting the impedance spectra recorded at 0.3 V vs. Ag/AgCl in 0.1 M BR buffer solution (pH 3.0) (Fig. 2B) to the equivalent circuit in Fig. 2C. The data relate to PBCP/GCE, PBCP/$AuNP_{chitosan}$/GCE and PBCP/$(AuNP+GQD)_{chitosan}$/GCE polymerization in ChCl:AcA:EG tDES at scan rate 50 mV s^{-1} with 1 M $HClO_4$ acid dopant.

GCE modifier	R_1 / Ω cm²	CPE_1 / µF cm^{-2} s$^{\alpha-1}$	α_1	Z_W / Ω cm²	α_{zw}	R_2 / Ω cm²	CPE_2 / mF cm^{-2}s$^{\alpha-1}$	α_2
PBCP	2.92 ± 0.68	0.023 ± 0.003	1 ± 0.28	1.09 ± 0.28	0.45 ± 0.01	2264 ± 1.7	0.085 ± 0.005	0.91 ± 0.04
PBCP/$AuNP_{chitosan}$	0.895 ± 0.31	0.454 ± 0.002	1 ± 0.30	1.22 ± 0.15	0.42 ± 0.03	2155 ± 1.3	0.059 ± 0.002	0.91 ± 0.01
PBCP/$(AuNP+GQD)_{chitosan}$	0.474 ± 0.22	1.022 ± 0.004	1 ± 0.12	1.27 ± 0.23	0.44 ± 0.05	2163 ± 2.1	0.065 ± 0.003	0.92 ± 0.02
